# Effectiveness of a broad-spectrum bivalent mRNA vaccine against SARS-CoV-2 variants in preclinical studies

**DOI:** 10.1080/22221751.2024.2321994

**Published:** 2024-02-20

**Authors:** Jing Lu, Shudan Tan, Hao Gu, Kunpeng Liu, Wei Huang, Zhaoli Yu, Guoliang Lu, Zihan Wu, Xiaobo Gao, Jinghua Zhao, Zongting Yao, Feng Yi, Yantao Yang, Hu Wang, Xue Hu, Mingqing Lu, Wei Li, Hui Zhou, Hang Yu, Chao Shan, Jinzhong Lin

**Affiliations:** a State Key Laboratory of Genetic Engineering, School of Life Sciences, Zhongshan Hospital, Fudan University, Shanghai, People’s Republic of China; b Shanghai Institute of Infectious Disease and Biosecurity, Fudan University, Shanghai, People’s Republic of China; c Center for mRNA Translational Research, Fudan University, Shanghai, People’s Republic of China; d Shanghai RNACure Biopharma Co., Ltd, Shanghai, People’s Republic of China; e State Key Laboratory of Virology, Wuhan Institute of Virology, Chinese Academy of Sciences, Wuhan, People’s Republic of China; f University of the Chinese Academy of Sciences, Beijing, People’s Republic of China; g Hubei Jiangxia Laboratory, Wuhan, People’s Republic of China

**Keywords:** RQ3025, COVID-19, bivalent mRNA Vaccine, SARS-CoV-2, Chimeric Spike, immune escape

## Abstract

Vaccines utilizing modified messenger RNA (mRNA) technology have shown robust protective efficacy against SARS-CoV-2 in humans. As the virus continues to evolve in both human and non-human hosts, risk remains that the performance of the vaccines can be compromised by new variants with strong immune escape abilities. Here we present preclinical characterizations of a novel bivalent mRNA vaccine RQ3025 for its safety and effectiveness in animal models. The mRNA sequence of the vaccine is designed to incorporate common mutations on the SARS-CoV-2 spike protein that have been discovered along the evolutionary paths of different variants. Broad-spectrum, high-titer neutralizing antibodies against multiple variants were induced in mice (BALB/c and K18-hACE2), hamsters and rats upon injections of RQ3025, demonstrating advantages over the monovalent mRNA vaccines. Effectiveness in protection against several newly emerged variants is also evident in RQ3025-vaccinated rats. Analysis of splenocytes derived cytokines in BALB/c mice suggested that a Th1-biased cellular immune response was induced by RQ3025. Histological analysis of multiple organs in rats following injection of a high dose of RQ3025 showed no evidence of pathological changes. This study proves the safety and effectiveness of RQ3025 as a broad-spectrum vaccine against SARS-CoV-2 variants in animal models and lays the foundation for its potential clinical application in the future.

## Introduction

In the fight against the coronavirus disease 2019 (COVID-19) pandemic, mRNA vaccines utilizing the lipid nanoparticle (LNP) delivery system have achieved remarkable success. Two mRNA vaccines, mRNA-1273 from Moderna and BNT162b2 from Pfizer-BioNTech, encoding the prefusion stabilized full-length spike (S) protein of SARS-CoV-2, are currently extensively employed and have demonstrated safety and effectiveness in preventing COVID-19 [1,2]. Shortly after the outbreak of the SARS-CoV-2 pandemic, numerous variants with mutations on the spike protein capable of conferring immunologic escape or increased transmissibility emerged, such as the alpha (B1.1.7), beta (B.1.351), gamma (P.1) and delta (B.1.617.2) variants [3–6]. In November 2021 and early 2022, the omicron (B.1.1.529) variant and its sublineages (BA.1, BA.2, BA.3, BA.4, BA.5) quickly became predominant in circulation due to their striking antibody evasion properties, leading to a significant reduction in the preexisting population-level immunity established by vaccination and prior infection [7–12]. Since then, a number of Omicron descendant sublineages have been identified and continue to dominate the current circulating variants of interest (VOIs) and variants under monitoring (VUMs), although none of the variants are currently classified as variants of concern (VOCs).

To counteract the immune escape abilities acquired by Omicron variants, bivalent mRNA vaccines have been developed and authorized for use in many countries, although the effectiveness of providing extra protection was shown to be moderate compared to the monovalent vaccines [13,14]. Nonetheless, the rapid emergence of novel variants presents challenges in vaccine development timelines, underscoring the need for the development of new vaccines capable of eliciting broadly neutralizing antibodies. Previously, we developed the mRNA vaccine candidate RQ3013, which demonstrated high effectiveness against the alpha and beta variants of SARS-CoV-2 [15,16]. An updated vaccine RQ3019 modified from RQ3013 was developed to target Omicron variants. Mutations identified in the spike (S) proteins of the Omicron BA.2, BA.4 and BA.5 variants are incorporated into the sequence of RQ3019, rendering cross-protective efficacy against various Omicron subvariants. In this study, we tested the combination of RQ3013 (B.1.1.7, B.1.351) and RQ3019 (BA.2, BA.4, BA.5) as a bivalent mRNA vaccine dubbed RQ3025 to overcome the antigenic divergence of SARS-CoV-2 variants. The immunogenicity, protective efficacy, and safety of RQ3025 were tested upon inoculation in murine, hamster, and rat models. The results demonstrate the broad neutralizing ability of RQ3025 against multiple SARS-CoV-2 variants in animal models, providing support for its clinical application.

## Materials and methods

### Facility and ethics statement

All experiments with live SARS-CoV-2 viruses were performed in the enhanced biosafety level 3 (P3+) facilities. All experiments with mice, hamsters, and rats were carried out in accordance with the Regulations in the Guide for the Care and Use of Laboratory Animals of the Ministry of Science and Technology of the People’s Republic of China.

The female K18-hACE2 Transgenic mice (6- to 8-week-old) and female hamsters (6- to 8-week-old) experiments with SARS-CoV-2 challenge were conducted under animal biosafety level 3 (ABSL3) facilities approved by the National Health Commission of the People’s Republic of China.

All animal procedures were approved by the Institutional Animal Care and Use Committee of the Institute of Medical Biology, Chinese Academy of Medical Science.

### Cell lines and antibodies

HEK 293F cells were grown in FreeStyle Media (Gibco-Thermo Fisher Scientific, NY, USA) and transfection was done by using Polyethylenimine Linear (PEI) MW40000 (Yeasen Biotechnology, Shanghai, China) in an 8% CO_2_ environment at 37°C. HEK 293 T and Vero E6 cells were maintained in high glucose DMEM (GIBCO) supplemented with 10% FBS (GIBCO) and 1% penicillin/streptomycin (P/S) (GIBCO) in 5% CO_2_ at 37°C.

The following primary antibodies were used in this study: SARS-CoV-2 Spike Protein (S1-NTD) (E7M5X) Mouse mAb #42172 (42172S, Cell Signaling, MA, USA) SARS-CoV-2 Spike Protein (S1) (E5S3 V) Rabbit mAb #99423 (99423S, Cell Signaling), SARS-CoV-2 (2019-nCoV) Spike S2 Antibody (40590-D001, Sino Biological, Beijing, China), SARS-CoV-2 (2019-nCoV) Spike Antibody (40589-T62, Sino Biological) and anti-GAPDH Antibody (60004, Proteintech, CA, USA). The secondary antibodies used were: Peroxidase AffiniPure Goat Anti-Rabbit IgG (H + L) (111-035-003, Jackson ImmunoResearch, PA, USA), Peroxidase AffiniPure Goat Anti-Mouse IgG (H + L) (115-035-003, Jackson ImmunoResearch), goat anti-Syrian hamster IgG HRP (ab6892, abcam, Cambridge, UK), Peroxidase AffiniPure Goat Anti-Rat IgG (whole molecule) (A9037, Sigma-Aldrich, MO, USA), Peroxidase AffiniPure Goat Anti-Human IgG, Fcγ fragment specific (109-035-098, Jackson ImmunoResearch), and HyperFluor 488 Goat Anti-Human IgG (H + L) Antibody (K1205, APExBIO, Shanghai, China).

### Virus titration

The virus titration of BA.5 followed this protocol. Endpoint titration in Vero E6 cells was employed for virus titrations. Vero E6 cells were seeded in a 96-well plate and incubated overnight at 37 °C. Subsequently, the cells were inoculated with 10-fold serial dilutions of the virus. One-hour post-inoculation, the inoculum was aspirated and replaced with 100 μL of DMEM (C11995500, Gibco, USA) supplemented with 2% FBS (10091148, Gibco) and 100 IU/mL penicillin–streptomycin (15140122, Gibco). After 4–7 days of incubation, the cytopathic effect was assessed, and the TCID_50_ was calculated.

The titration of the Beta virus for the novel coronavirus variants was determined utilizing the Focus Forming Assay (FFA). This method involves immunostaining Vero E6 cells infected with the virus, utilizing an anti-SARS-CoV-2 N-protein antibody along with an HRP-conjugated secondary antibody. Subsequently, the stained spots on cell plates were quantified and analyzed using an enzyme-linked immunospot analyzer (ELISPOT device). The titer (FFU/mL) was calculated using the formula: titer (FFU/mL) = number of spots × dilution factor / volume, with the detection limit determined when one spot was observed in the undiluted experimental sample.

### Preparation of modified mRNAs and LNP

Production of RQ3013, RQ3019 and RQ3025 mRNA and LNP used in this study was conducted under current good manufacturing practice (cGMP). The DNA templates for RQ3013 and RQ3019 RNA were cloned into the plasmid with backbone elements (T7 promoter, 5′ and 3′ UTR, poly(A) tail). RQ3013 and RQ3019 mRNA was in vitro-transcribed by T7 RNA polymerase in the presence of the Cap1 analog (B8176, APExBIO) and nucleotides with a global substitution of uridine with pseudouridine (Ψ, APExBIO). RNA was purified through two chromatographic procedures. RNA integrity was assessed by microfluidic capillary electrophoresis, and the concentration, pH, osmolality, and endotoxin level were also measured for quality control. The purified RNA was then formulated into lipid nanoparticles (LNPs) containing four lipids (an ionizable lipid, PEG2000-DMG, DSPC, and cholesterol) at a pilot scale. The resulting nanoparticles were further purified using tangential flow filtration. Sucrose was added as a cryoprotectant, and the solution was adjusted to reach a concentration of 200 μg/mL (mRNA drug substance) before being sterilized by filtration. The vials were then filled with the formulated LNP and stored at –20 °C until further use. The final product was characterized for particle size, polydispersity, encapsulation efficiency, zeta potential, mRNA purity, double-stranded RNA content, osmolality, pH, endotoxin, and bioburden.

### Western blot analysis of RQ3013, RQ3019 and RQ3025-LNP transfected cells

The protein expressions of mRNA-LNPs were tested in HEK 293 T cells. Five μg of mRNA-LNPs were added to 5 × 10^5^ HEK 293 T cells in one well of a 12-well plate and incubated for 18 h. Briefly, cells were rinsed with PBS and lysed in lysis buffer (20 mM Tris-HCl pH 7.4, 150 mM NaCl, 3 mM MgCl_2_, 1% Triton X-100) supplemented with protease inhibitor (APExBIO). The cell lysates were centrifuged at 14,000 rpm for 20 min and supernatants were mixed with SDS loading buffer. The protein samples were separated in a 4–20% gradient SDS-Gel, and transferred to PVDF membranes (ThermoFisher, WM, USA) by Trans-Blot Turbo Transfer System (BioRad, CA, USA). The membranes were blocked with 5% non-fat dry milk in PBS with 0.1% Triton X-100 and then incubated with primary antibodies and subsequently with HRP-conjugated secondary antibodies with three washing steps after each incubation. Signals were detected with an enhanced chemiluminescence (ECL) detection system (APExBIO).

### Localization of expressed vaccine antigens by immunofluorescence

Cells transfected with LNP-mRNA were cultured for 18–24 h and fixed in 4% paraformaldehyde (PFA) for 15 min, followed by three washes with cold PBS. Fixed cells were blocked in 1% bovine serum albumin (BSA) in PBS for 30 min at room temperature and incubated with primary and fluorophore-conjugated secondary antibodies diluted in PBS for 1 h at 37°C with three washes in PBS after each incubation. Nuclei were co-stained by incubation with 4,6-diamidino-2-phenylindole dihydrochloride (DAPI) for 5 min followed by three washes in PBS. Images were acquired using Leica DMi8 microscope.

### Cloning, expression, and preparation of the RQ3013 and RQ3019 encoded Spike proteins

The DNA fragments encoding RQ3013 or RQ3019 were fused with a C-terminal twin Strep-tag (LEVLFQGPSGS WSHPQFEK GGGSGGGSGGSA WSHPQFEK) and cloned into a mammalian cell expression vector pcDNA3.1. The resulting plasmid, pcDNA3.1-RQ3013-Twinstrep and pcDNA3.1-RQ3019-Twinstrep, were transfected into HEK 293F cells using polyethylenimine (PEI) in FreeStyle Media (Gibco) for the purification of the RQ3013 and RQ3019 proteins, respectively.

To purify the full-length S protein, the transfected cells were harvested at a density of ∼4–5 × 10^6^/mL by centrifugation (1000 × g at 4°C for 30 min). The cell pellet was washed with cold PBS, resuspended in a cold lysis buffer containing Buffer A (100 mM Tris-HCl, pH 8.0, 150 mM NaCl, 1 mM EDTA) and 1% (w/v) n-dodecyl-β-D-maltopyranoside (DDM) (Anatrace, Inc. Maumee, OH), 1 × EDTA-free complete protease inhibitor cocktail (APExBIO), and incubated at 4°C for 1 h.

The protein purification was conducted as described. Briefly, after clarifying spin at 30,000 × g for 60 min at 4°C, the supernatant was loaded on a strep-tactin (IBA Lifesciences, Germany) column equilibrated with the lysis buffer. The column was washed with 50 column volumes (CV) of Buffer A and 0.3% DDM, followed by 50 CV Buffer A and 0.1% DDM, and 50CV Buffer A and 0.02% DDM. The S protein was eluted by Buffer A containing 0.02% DDM and 50 mM biotin and further purified by gel filtration chromatography on a Superose 6 10/300 column in a buffer containing 25 mM Tris-HCl, pH 7.5, 150 mM NaCl, and 0.02% DDM.

### Animal vaccination and serum collection

Prior to vaccination, vaccine vials were thawed at 4°C for 30 min and then diluted to the desired concentrations using saline. For mice, RQ3013, RQ3019 or RQ3025 was diluted to 40 and 100 μg/mL for 2 and 5 μg doses, respectively. For hamsters, the vaccine was diluted to 33 and 166 μg/mL for 5 and 25 μg doses, respectively. Rats received the vaccine without dilution.

#### Mice

For mouse vaccination, groups of 6- to 8-week-old female BALB/c mice or female K18-hACE2 mice were intramuscularly immunized with LNP vaccine candidates or control in 50 μL volume, and 3 weeks later, a second dose was administered to boost the immune responses.

#### Hamsters

6- to 8-week-old female hamsters were immunized intramuscularly with 150 μL of LNP vaccine candidates or control at week 0. At week 3, all hamsters received a boost vaccination.

#### Rats

Sprague–Dawley rats, male or female, aged between 6 and 8 weeks old, were divided into five groups (*n* = 10/group, 5 males and 5 females) and immunized via intramuscular injection, with a two-week interval between doses. The five groups received a high dose (40 μg/injection) or low dose (10 μg/injection) vaccine, high dose or low dose empty LNP only, or saline. Each animal was administered three doses.

Blood samples from mice, hamsters, and rats were collected by retro-orbital bleeding and inactivated at 56°C for 0.5 h prior to measurements of SARS-CoV-2-specific IgG and neutralizing antibodies.

### ELISA for SARS-CoV-2 S-specific IgG

SARS-CoV-2 S-specific antibody responses in immunized sera were determined by enzyme-linked immunosorbent assay (ELISA) as previously described [15]. Briefly, 96-well plates were coated with 50 μL of coating buffer containing 100 ng/well recombinant SARS-CoV-2 spike, S2 subunit or RBD antigens (Sino Biological, Beijing, China) at 4°C overnight. Plates were blocked with 2% BSA in PBS containing 0.1% Triton X-100 at room temperature for 1 h. Immunized mice sera were diluted 100-fold from the initial concentration, and then a 5-fold serial dilution of a total of 11 gradients in PBS. Serially diluted sera were then added to the plates and incubated at room temperature for 2 h. Plates were incubated with goat anti-mouse IgG HRP (for mouse sera, Proteintech Cat: SA00001-1, USA) or goat anti-Syrian hamster IgG HRP (for hamster sera, Abcam Cat: ab6892) or goat anti-Rat IgG HRP (for rat sera, SIGMA Cat: A9037-1ML, Germany) at 37°C for 1 h and then substrate tetramethylbenzidine (TMB) solution (Invitrogen, CA, USA) was used to develop. The color reaction was quenched with 1N sulfuric acid for 10 min, and the optical density was measured at 450 nm wavelength by VARIOSKAN LUX (Thermo Fisher).

### Pseudovirus-based neutralization assay

The neutralization assay was conducted using pseudovirus particles. For the production of pseudovirus, 293 T cells were co-transfected with three plasmids, namely pMD2.G, pSPAX2, and an expression plasmid encoding the codon-optimized full-length spike protein derived from the wild-type (WT) COVID-19 strain or other mutant strains. In brief, 6 × 10^6^ 293 T cells were seeded in a 100 mm culture dish one day prior to transfection. On the following day, the cells were co-transfected with 5 μg of each plasmid (totaling 15 μg). Pseudoviruses were harvested from the culture supernatant at 48 and 72 h post-transfection, and subsequently stored at −70 °C in 1.0 mL aliquots until further utilization. Serum samples obtained from immunized animals were serially diluted with cell culture medium and incubated with the pseudovirus suspension at 1:1 ratio in 96-well plates for 1 h at room temperature. Next, Opti-HEK293/ACE2 cells were added to the serum-virus mixture, and the plates were incubated at 37°C with 5% CO_2_ for 48 h. The degree of SARS-CoV-2 pseudovirus infection was assessed by measuring the luciferase activity using luciferase assay (PerkinElmer, MA, USA). Neutralizing Titer 50% (NT_50_) was determined as the highest serum dilution that inhibited pseudovirus infection by more than 50% relative to the control group. Non-linear regression analysis was carried out on GraphPad Prism 8.0 using the 4-parameter logistic sigmoidal dose–response model to calculate the NT_50_ value.

### SARS-CoV-2 neutralization assay

#### Plaque reduction neutralization test (PRNT)

One day before the neutralization assay, 2 × 10^5^ Vero E6 cells per well were seeded in a 24-well cell culture plate to form a dense monolayer of cells on the day of the assay. Serum samples were heat-inactivated at 56°C for 60 min, diluted serially as 1:50, 1:150, 1:450, 1:1350, 1:4050, and 1:12150 in sample dilution buffer and then mixed with diluted virus (1000 pfu/mL) in equal volume and incubated at 37°C for 1 h. The “diluted test sample-neutralizing virus mixture” was added to the 24-well cell culture plate after removing the culture medium and incubated at 37°C for 1 h with agitation every 15 min. The supernatant was removed and 0.8% methylcellulose maintenance medium (0.5 mL/well) was added and the plate was incubated at 37°C for 4 days. For control, a sample dilution buffer without adding serum samples was used in the same procedure performed as above. After 4 days incubation, 8% paraformaldehyde (PFA) solution (0.5 mL/well) was added to the plate and incubated at room temperature for 1 h. Crystal violet (0.5%, 0.25 mL/well) was added for staining after removing PFA. The plaques were counted for calculating the titer. Non-linear regression analysis was conducted using GraphPad Prism8.0 with the 4-parameter logistic sigmoidal dose–response model to calculate the Inhibitory Concentration 50% (IC_50_).

#### Focus forming assay (FFA)

First an antibody serial dilution plate as prepared by diluting the antibodies in DMEM without FBS, starting from 1:8 in 25 μL volume per well in a 96-well cell culture plate. Next, 25 μL of live virus dilution (containing 100–300 FFU live virus) was added to each well and mixed thoroughly, followed by incubation at 37°C with 5% CO_2_ for 1 h. The antibody-virus mixture (50 µL/well) was then added to the 96-well cell culture plates pre-seeded with Vero E6 cells (2 × 10^4^ cells per well) and incubated at 37°C with 5% CO_2_ for 1 h. After incubation, the supernatant was replaced by 100 μL of 1.6% CMC culture medium and cells were continue incubated at 37°C and 5% CO_2_ for 24 h. Cells were then fixed with 300 μL of 4% PFA for 1 h and washed with PBS, followed by permeabilization in PBS with 0.2% TritonX-100 and blocking with 10% BSA in PBS. Subsequently, 50 μL of diluted primary and secondary antibodies were added to each well and incubated at 37°C for 1 h with PBST washing steps following each incubation. Next, KPL True-blue chromogenic solution was added (50 µL/well) and incubated at room temperature in the dark for 10 min followed by washing. The plate was then air-dried, and the plate spots were counted using an enzyme-linked immunospot analyzer. The inhibition rate was calculated as 100% × (1 − sample spot count / positive control spot count).

FRNT_50_ was calculated as the logarithm of the virus dilution that caused more than 50% inhibition plus the ratio distance multiplied by the difference between the logarithm of the virus dilution that caused less than 50% inhibition and the logarithm of the virus dilution that caused more than 50% inhibition. Where: Ratio distance = (Spot count of 50% inhibition in the positive control well – Spot count of the virus dilution causing more than 50% inhibition) / (Spot count of the virus dilution causing less than 50% inhibition – Spot count of the virus dilution causing more than 50% inhibition). The corresponding antilogarithm represents the FRNT_50_ value.

### Intracellular cytokine staining assay

For the intracellular cytokine staining, mouse spleen cells were stimulated with 2 μg/mL S1, S2 peptide pools spanning SARS-CoV-2 spike S1 and S2 respectively (15mers, overlapping by 11aa, GenScript, Nanjing, China) or equimolar amount of DMSO (negative control) in the presence of anti-mouse CD28 antibody (BD Bioscience, CA, USA) for 1 h. GolgiStop protein transport inhibitor (BD Bioscience) was added into the culture and further incubated for 5 h. After stimulation, cells were washed and stained with Fixable Viability Stain 510 (BD Bioscience). Cells were then blocked with anti-mouse CD16/32 and labeled with cell surface antibody cocktail including anti-mouse CD3-FITC, anti-mouse CD4-APC and anti-mouse CD8-Percp-cy5.5 (BD Bioscience) for 30 min at 4°C. Following surface staining, cells were incubated with fixing/permeabilizing solution (BD Bioscience) for 20 min at 4°C and stained with intracellular antibodies including anti-mouse IFN-γ-Pe-Cy7, IL-2-BV605, TNF-α-BV650, IL-4-BV711, and IL-5-PE (BD Bioscience) for 30 min at 4°C. Cells were washed and resuspended in PBS buffer and analyzed on a CYTEK Aurora/NL.

### IFN-γ and IL-2 ELISpot assays

The number of spleen cells secreting IFN-γ and IL-2 was detected by enzyme-linked immunospot (ELISpot) assays. In brief, single cell suspension from the mouse spleen was prepared and stimulated with 2 μg/mL spike S1, S2 peptide pool or equimolar amount of DMSO (negative control) for 22 h. IFN-γ and IL-2-secreting cells were detected using ELISpot kits (DAKEWE, Shenzhen, China and MABTECH, Stockholm, Sweden) according to the manufacture protocol and spots were counted using an ELISpot Analyzer AT-SPOT (SINSAGE, Beijing, China).

### Spleen cell stimulation and cytokine detection

Single cell suspension was prepared from mouse spleen and 2 × 10^6^ spleen cells were stimulated with 1 μg/mL peptides pool of spike S1 and S2 or equimolar amount of DMSO (negative control) for 22 h at 37°C. Supernatant was collected following incubation and frozen at −80°C for cytokine detection. Cytokine in the supernatant was measured with a V-PLEX Proinflammatory Panel 1 Mouse Kit (Meso Scale Discovery, FL, USA) using Meso QuickPles SQ 120 (Meso Scale Discovery).

### Animal challenge experiments

#### K18-hACE2 transgenic mice

Mice were immunized with RQ3025 (2 and 5 µg) or saline on day 0 and 21. K18-hACE2 mice vaccinated with RQ3025 or saline were challenged intranasally with the variant B.1.351 (1 × 10^3^ PFU) 42 days post-boost or BA.5 (0.7 × 10^5^ PFU) 23 days poost-boost. The body weights of the mice were recorded daily. At 4 or 5 dpi, all mice were sacrificed and the right lung, trachea and brain were collected for viral load detection. Left lung tissue was collected for histopathological assay.

#### Hamsters

Hamsters (6- to 8-week-old) were divided into three groups and injected intramuscularly with high dose (25 μg/dose), low dose (5 μg/dose) vaccine, and saline respectively. All animals were immunized twice (days 0 and 21) before being intranasally challenged with 0.7 × 10^5^ PFU BA.5 SARS-CoV-2 virus. The body weights of the hamsters were recorded daily. Hamsters were euthanized, and lung tissues were collected at 5 dpi for RT-qPCR and histopathological assays.

### RT-qPCR

The quantification of viral load in the tissues of K18 mice or hamsters following the challenge with the BA.5 variant was conducted using the following method. Virus RNA quantification in the samples was performed using one-step real-time quantitative RT-qPCR technique following the supplier's instructions. Tissues were homogenized in DMEM (1:10, W/V), centrifuged at 4500 g for 30 min at 4°C, and the supernatants were collected for RNA extraction using an automated nucleic acid extraction and purification system (GENFINE, Beijing, China). RNA was eluted in 100 μL of elution buffer and used as the template for RT–PCR. The HiScript® II One Step qRT-PCR SYBR® Green Kit (Vazyme Biotech Co., Nanjing, China) was employed for determining the RNA copy number in the samples. The RT-qPCR was conducted on a Bio-Rad CFX96 Touch Real-Time PCR Detection System with cycling program of 50°C for 3 min and 95°C for 30 s, followed by 40 cycles of 95°C for 10 s and 60°C for 30 s. The default melting curve was obtained, and based on the standard curve, the virus copy number was calculated (Copies/mL). Primers targeting the virus spike gene used were: RBD-qF1: 5′-CAATGGTTAAGGCAGG-3′; RBD-qR1: 5′-CTCAAGGTCTGGATCACG-3′.

The viral load in the tissues of K18 mice post-challenge with the Beta variant was determined using a qPCR assay kit (DA0992). A standard curve was established using the SARS-CoV-2-N gene standard, and the relationship between CT values and copy numbers was calculated by qPCR software. Detailed procedural steps can be found in the instructions for the “Novel Coronavirus 2019-nCoV Nucleic Acid Detection Kit (Fluorescent PCR Method).”

### Histopathology and immunohistochemistry

The experimental procedures for the pathological analysis of lung tissues in K18 mice or hamsters post-challenge with the BA.5 variant are as follows. Animals were anesthetized by isoflurane inhalation and euthanized by exsanguination through abdominal aorta. Tissues were dissected and fixed in 4% paraformaldehyde solution for 7 days, dehydrated in rising series of ethanol and embedded in paraffin. Hematoxylin and eosin (HE) staining was performed on 3 µm thick paraffin sections. Images were acquired using an optical microscope (Nikon Eclipse Ci) equipped with camera (Nikon DS-Fi2).

The experimental procedures for the pathological analysis of lung tissues in K18 mice post-challenge with the Beta variant are outlined as follows. Four days post-challenge with the virus, K18-hACE2 transgenic mice were humanely euthanized, and their lungs were dissected. Individual lung lobes were weighed and then fixed in 4% paraformaldehyde and processed routinely into paraffin blocks. Tissue sections were prepared, and slides were stained with hematoxylin and eosin (H&E) before examination by microscopy imaging system (LEICA DM3000). Slides were scored from 0 (no pathology) to 4 (severe pathology) for alveolar wall necrosis, alveolitis and perivasculitis, fibrin exudation, and endothelial proliferation or evaluated for inflammation area (%) by a blinded pathologist.

### Vaccine safety evaluation

Five groups of Sprague–Dawley rats (5 female and 5 male rats/group) were immunized with high dose (40 μg) or low dose (10 μg) vaccine; high dose or low dose empty LNP; and saline individually for three times on days 0, 14 and 28. A panel of safety-related parameters were measured during and after immunization, including clinical observation, body weight and body temperature. Blood samples were collected from animals at 3 days after the last dose (day 31) and after the recovery period (day 42) for lymphocyte subset analysis and cytokine measurements. Lymphocyte subpopulations, including CD3^+^, CD3 ^+ ^CD4^+^, and CD3 ^+ ^CD8^+^ cells, were determined by FACS (BD FACSCanto^TM^ II). TNF-α, IFN-γ, IL-2, and IL-6 levels were detected using a commercial kit (BioLegend, CA, USA) and analyzed by FACS (BD LSRFortessa^TM^). Blood biochemical indices were measured using TBA-120FR automatic biochemical analyzer. 60% of rats were euthanized at day 31 post-immunization, and the rest were euthanized on day 42. Organs of lung, brain, heart, liver, spleen, and kidney were collected for pathologic analysis.

### Statistical analysis

Statistical analysis was conducted using Graphpad Prism software, version 8.00. NT_50_ determination was performed using a log (inhibitor) vs. response – Variable slope (four parameters) test, and correlation analysis was conducted using linear regression (Pearson analysis). Multiple comparisons were analyzed using one-way or two-way ANOVA and Tukey’s multiple comparison tests. *P*-values less than 0.05 were considered statistically significant and denoted as follows: *P < 0.05, **P < 0.01, ***P < 0.001, ****P < 0.0001.

## Supplementary Material

Supplementary_Information_sent

Supplementary_Information_sent

## Figures and Tables

**Figure 1.**
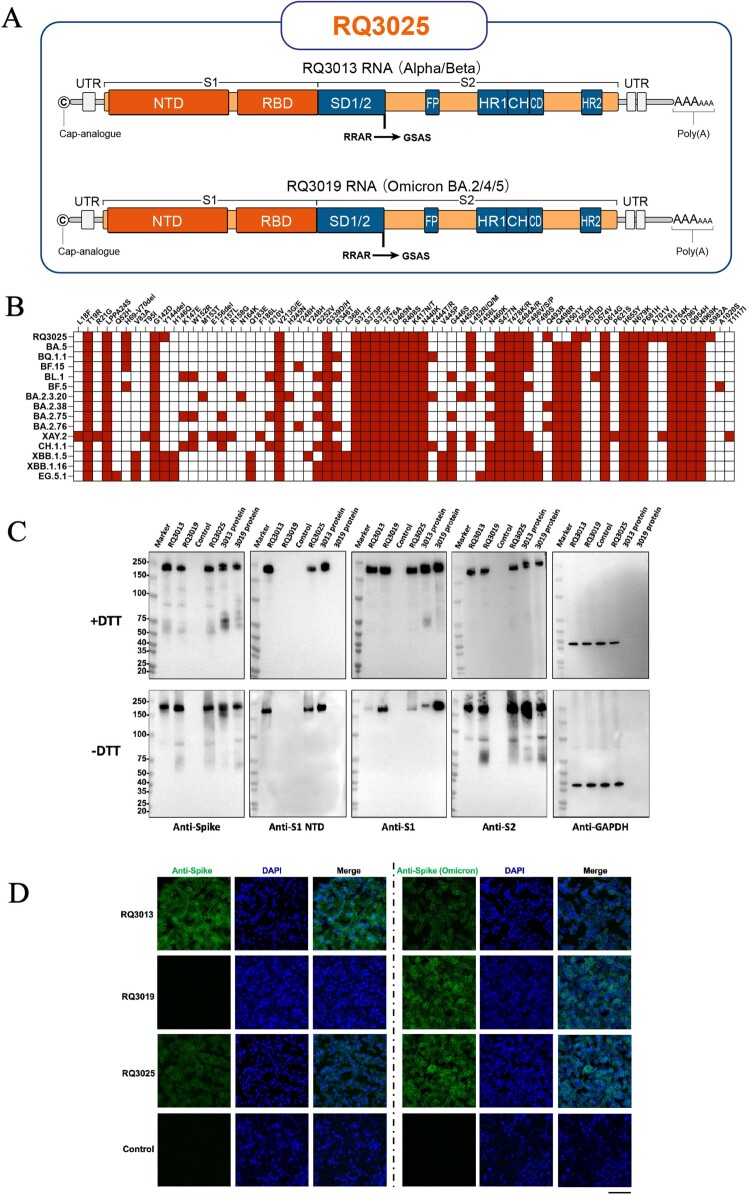
Vaccine design of RQ3025 and structural characterization of the expressed antigens. (A) An illustration of the composition of the RQ3025 Bivalent Vaccine, containing two mRNA sequences (RQ3013 and RQ3019) encoding the Spike protein of SARS-CoV-2. The furin cleavage site RRAR was replaced by GSAS.The last 18 amino acids were deleted. (B) Heatmap analysis of the mutation sites of the bivalent mRNA vaccine RQ3025 and the mutation sites of prevalent variant strains. (C) Western blots using different spike protein antibodies showing specificities of the antigens expressed by HEK293 T cells transfected with RQ3013, RQ3019, RQ3025, and Control. Purified RQ3013 and RQ3019 proteins were run in parallel on the same gels as positive control. (D) Localizations of the RQ3025, RQ3013 and RQ3019 RNA-encoded Spikes in HEK293 T cells transfected with the vaccine constructs were determined by immunofluorescence. Scale bar, 100 μm.

**Figure 2.**
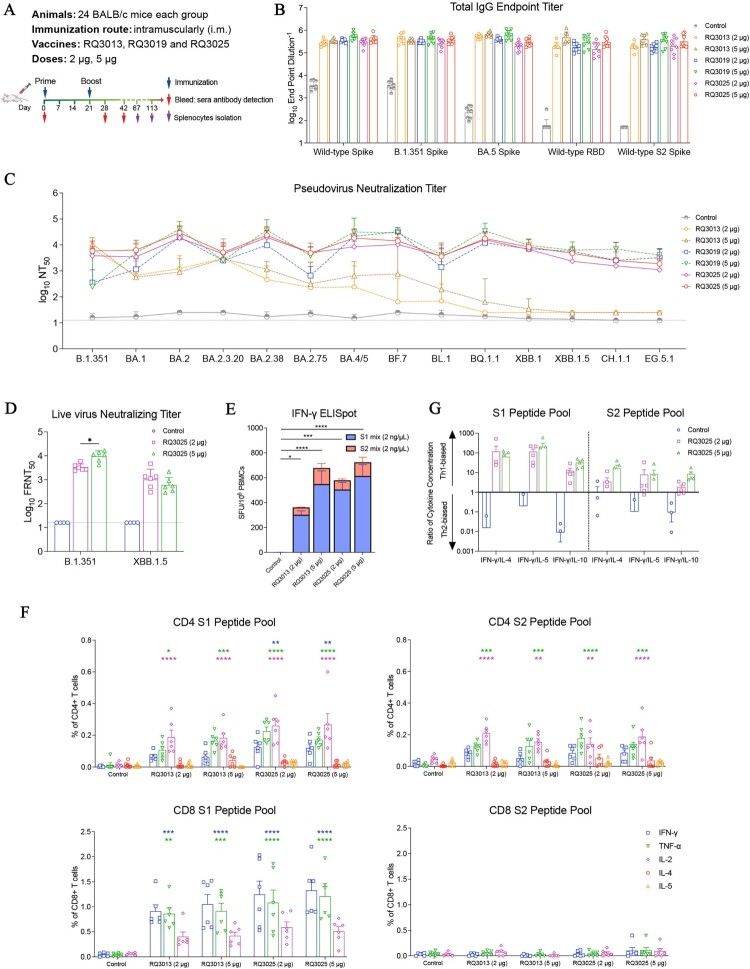
Immunogenicity of RQ3025 in BALB/c mice. (A) The immunization scheme for RQ3013, RQ3019 and RQ3025 in mice. Mice (*n* = 24 per group) were intramuscularly immunized with saline (Control), low dose (2 μg) or high dose (5 μg) of RQ3013, RQ3019 or RQ3025. Time points of vaccination, bleeding, and splenocyte isolation are indicated by arrows. Eight or six mice from each group were randomly selected for ELISA or neutralizing activity measurement, respectively, while the rest were used to detect the T cell responses. (B) IgG antibody responses were measured by analyzing the binding of antibodies to the wild-type RBD antigen, S proteins from the wild-type and SARS-CoV-2 variants, and the wild-type S2 subunit using sera collected on day 28 via enzyme-linked immunosorbent assay (ELISA). Values are geometric mean ± SD. (C) Neutralizing antibody titers in sera collected on day 28, analyzed by the lentiviral luciferase-based pseudovirus assay. The black dashed line indicates the assay's detection limit (reciprocal titer of 12.5). Values are geometric mean ± SD. (D) The FFA-based live virus micro-neutralization assay of week-6 sera against SARS-CoV-2 B.1.351 and XBB.1.5. The black dashed line indicates the assay's detection limit (reciprocal titer of 16). Values are geometric mean ± SD. (E) Summed IFN-γ ELISpot responses in splenocytes restimulated with peptides spanning the entire S protein. The splenocytes were collected on day 113. SFU, spot-forming units. Data are presented as mean ± SEM. (F) CD4^+^ and CD8^+^ T cell responses in isolated splenocytes at 92 days post-boost stimulated with either vehicle or pools of overlapping peptides derived from the SARS-CoV-2 S protein for 5 h, shown by flow cytometry analyses of IFN-γ, IL-2, TNF-α, IL-4 and IL-5 expressions. Background cytokine expression in the presence of vehicle alone was accounted for and subtracted from the measured responses elicited by the S1 and S2 peptide pools for each individual mouse. Data are presented as mean ± SEM. The significance analysis was conducted in comparison with the control group. (G) MSD assay showing IFN-γ, IL-4, IL-5, and IL-10 cytokine levels secreted by T cells restimulated with S1 and S2 peptide pools in isolated splenocytes 46 days post-boost. Data are presented as mean ± SEM. Statistical analyses were carried out by ANOVA and Tukey’s multiple comparison tests (*P < 0.05; **P < 0.005; ***P < 0.001; ****P < 0.0001).

**Figure 3.**
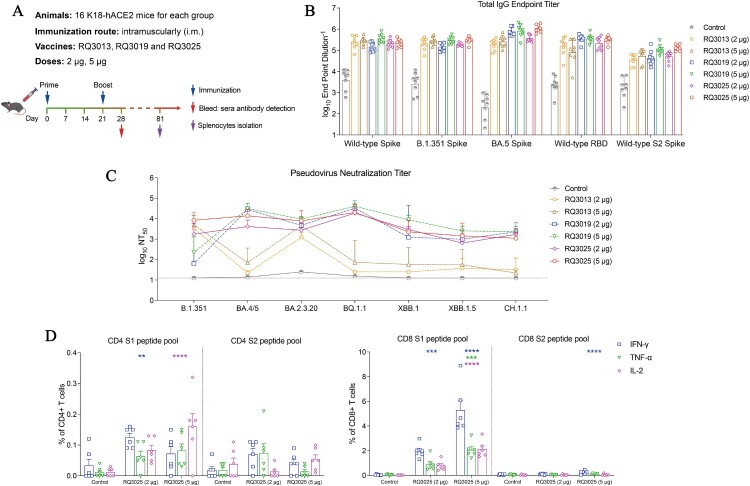
Immunogenicity of RQ3025 in K18-hACE2 transgenic mice. (A) The scheme of mice immunization. Mice were intramuscularly immunized with saline (Control), low dose (2 μg) or high dose (5 μg) of RQ3013, RQ3019 or RQ3025 (*n* = 16 per group). Time points of vaccination, bleeding, and splenocyte isolation are indicated by arrows. (B) ELISA analysis of IgGs binding to the wild-type RBD antigen, S proteins from the wild-type and SARS-CoV-2 variants, and the wild-type S2 subunit with sera collected on day 28. Values are geometric mean ± SD. (C) Neutralizing antibody titers in day-28 sera, analyzed by the lentiviral luciferase-based pseudovirus entry assay. The black dashed line indicates the detection limit of the assay (reciprocal titer of 12.5). Values are geometric mean ± SD. (D) The percentages of CD4^+^ and CD8^+^ T cells expressing IFN-γ, IL-2, and TNF-α were assessed using intracellular cytokine staining and flow cytometry in splenocytes collected 60 days after the second immunization and stimulated with a pool of peptides derived from either the S1 or S2 regions of the SARS-CoV-2 Spike protein. Data are presented as mean ± SEM. The significance analysis was conducted in comparison with the control group. Statistical analyses were carried out by ANOVA and Tukey’s multiple comparison tests (**P < 0.005; ***P < 0.001; ****P < 0.0001).

**Figure 4.**
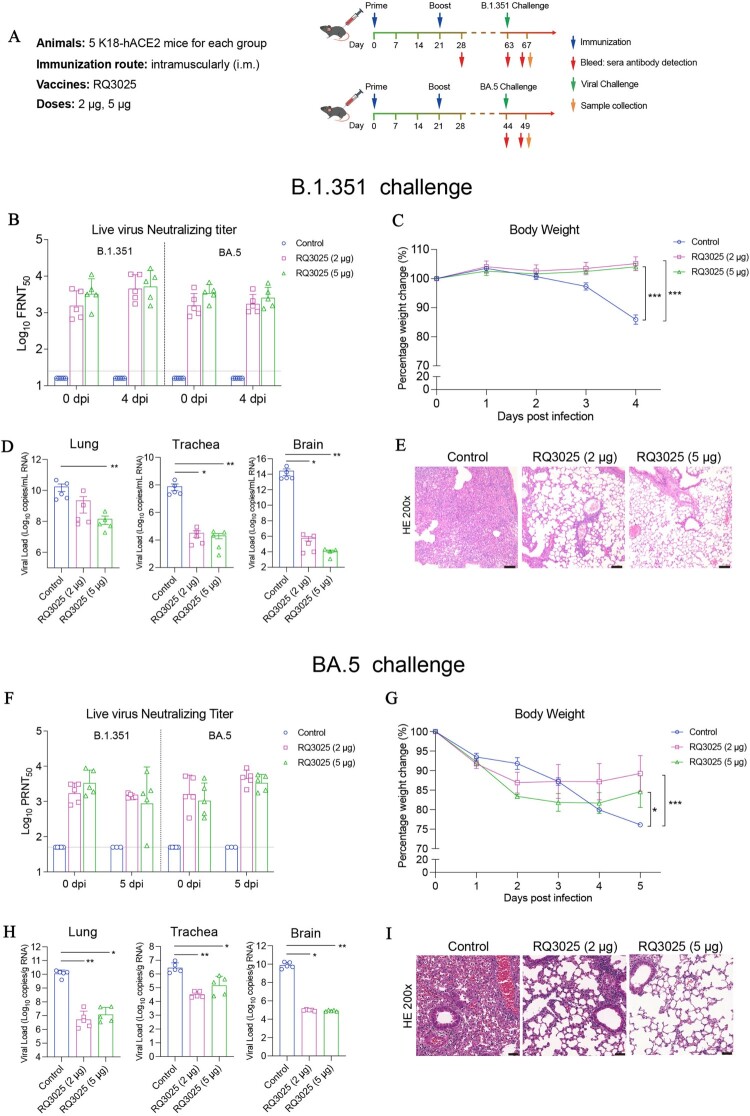
Protective efficacy of RQ3025 in K18-hACE2 transgenic mice. (A) The scheme of mice immunization. Mice were intramuscularly immunized with saline (Control) or low dose (2 μg) or high dose (5 μg) of RQ3025 (*n* = 5 per group). Time points of vaccination, viral challenge, and sample collection are indicated by arrows. Data collected from mice challenged with B.1.351 are shown in (B-E). (B) Live virus neutralization titers at 0 dpi and 4 dpi were detected by the FFA-based live virus microneutralization assay. The black dashed line indicates the detection limit of the assay (reciprocal titer of 16). Values are geometric mean ± SD. (C) Monitoring of body weight changes in K18-hACE2 mice following infection with the variant B.1.351. Data are presented as mean ± SEM. (D) Viral load in the lungs, tracheae and brains at 5 dpi was determined by RT-qPCR. Data are presented as geometric mean ± SD. (E) Histopathological examinations (hematoxylin and eosin (H&E) staining) of lungs from the challenged mice at 5 dpi. Scale bar, 100 μm. Data collected from mice challenged with BA.5 are shown in (F-I). (F) Live virus neutralization titers at 0 and 5 dpi were detected by the PRNT-based neutralization assay. The black dashed line indicates the detection limit of the assay (reciprocal titer of 50). Values are geometric mean ± SD. (G) Monitoring of body weight changes in K18-hACE2 mice following infection with the variant BA.5. Data are presented as mean ± SEM. (H) Viral load in the lung, trachea and brain tissues at 5 dpi was determined by RT-qPCR. Data are presented as geometric mean ± SD. (I) Histopathological examinations (H&E staining) of the lungs from infected mice at 5 dpi. Scale bar, 100 μm. Statistical analyses were carried out by ANOVA and Tukey’s multiple comparison tests (*P < 0.05; **P < 0.005; ***P < 0.001).

**Figure 5.**
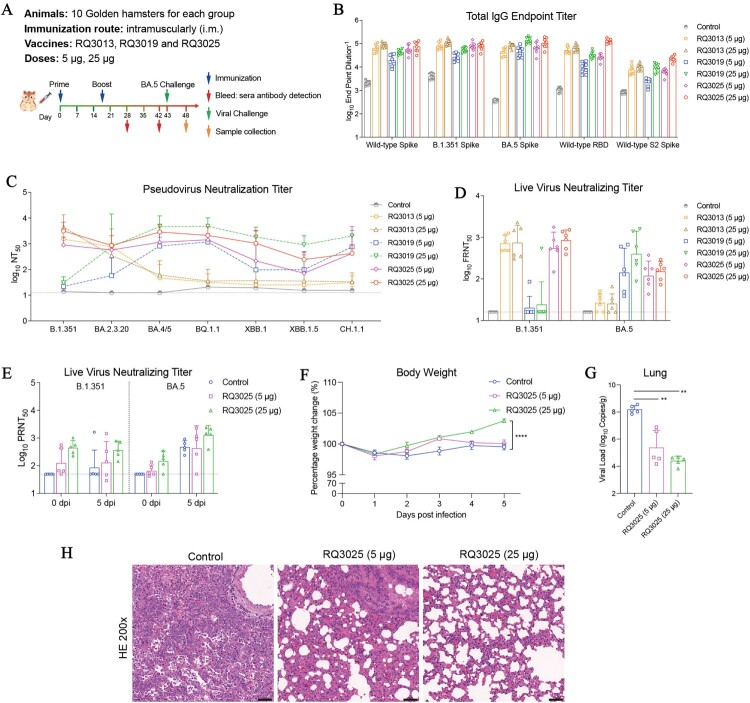
Immunogenicity and protective efficacy of RQ3025 in hamsters. (A) The immunization scheme for hamsters by RQ3013, RQ3019 and RQ3025. Hamsters were intramuscularly immunized with saline (Control), low dose (5 μg) or high dose (25 μg) of RQ3013, RQ3019 or RQ3025 (*n* = 10 per group). Time points of vaccination, bleeding, viral challenge, and sample collection are indicated by arrows. (B) Binding of IgGs to the wild-type RBD, S proteins from the wild-type and SARS-CoV-2 variants and the wild-type S2 subunit analyzed with sera collected on day 28 by ELISA. Values are geometric mean ± SD. (C) Neutralizing antibody titers in day-28 sera analyzed by the lentiviral luciferase-based pseudovirus assay. The black dashed line indicates the assay's limit of detection (reciprocal titer of 12.5). Values are geometric mean ± SD. (D) The FFA-based live virus microneutralization assay of week-6 sera against SARS-CoV-2 B.1.351 and BA.5. The black dashed line indicates the assay's detection limit (reciprocal titer of 16). Values are geometric mean ± SD. (E) Live virus neutralization titers at 0 and 5 dpi were detected by the PRNT-based neutralization assay. The black dashed line indicates the detection limit of the assay (reciprocal titer of 50). Values are geometric mean ± SD. (F) Body weight changes of hamsters following infection with the variant BA.5. Data are presented as mean ± SEM. (G) Viral load in the lungs at 5 dpi was determined by RT-qPCR. Data are presented as geometric mean ± SD. (H) Histopathological examinations (H&E staining) of the lungs from infected hamsters at 5 dpi. Black scale bar, 100 μm. Statistical analyses were carried out by ANOVA and Tukey’s multiple comparison tests (**P < 0.005; ****P < 0.0001).

**Figure 6.**
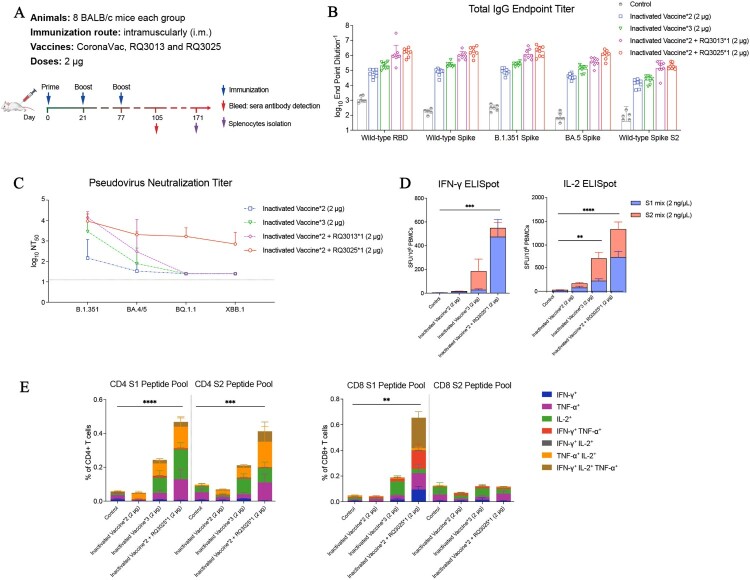
Immunogenicity of RQ3025 administered as a third booster in BALB/c mice previously received the inactivated vaccine. (A) The immunization scheme for the vaccines. BALB/c mice (*n* = 8 per group) were vaccinated on days 0 and 21 through the intramuscular route with a SARS-CoV-2 inactivated vaccine, and administered a booster dose of either the inactivated vaccine, RQ3013 or RQ3025 on day 77. Time points of vaccination, bleeding and splenocyte isolation are indicated by arrows. (B) Binding of IgGs to the wild-type RBD, S proteins from the wild-type and SARS-CoV-2 variants and the wild-type S2 subunit were analyzed with sera collected 4 weeks post-boost (on day 105) by ELISA. Values are geometric mean ± SD. (C) Neutralizing antibody titers in sera collected on day 105, analyzed by the lentiviral luciferase-based pseudovirus assay. The black dashed line indicates the assay's detection limit (reciprocal titer of 12.5). Values are geometric mean ± SD. (D) IFN-γ and IL-2 ELISpot assays on splenocytes collected 94 days after the third booster immunization (day 171) and stimulated with overlapping peptide pools derived from the S1 and S2 domains of the Spike protein. SFU, spot-forming units. Data are presented as mean ± SEM. (E) Flow cytometry analysis showing the percentages of CD4^+^ and CD8^+^ T cells expressing IFN-γ, IL-2 or/and TNF-α in splenocytes harvested 94 days after the last boost immunization (day 171) and stimulated with a pool of peptides derived from the S1 or S2 regions of the SARS-CoV-2 Spike protein, respectively. Data are represented as mean ± SEM. Statistical analyses were carried out by ANOVA and Tukey’s multiple comparison tests (**P < 0.005; ***P < 0.001; ****P < 0.0001).

**Figure 7.**
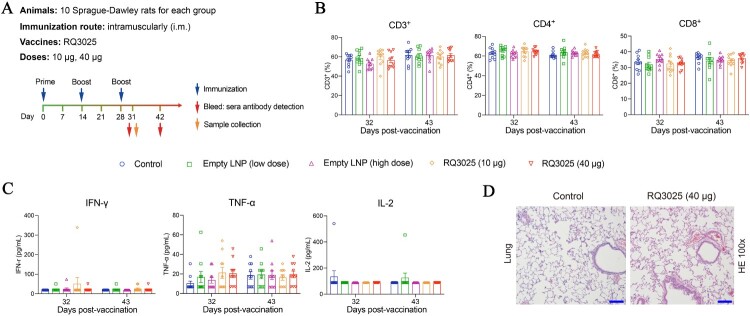
Safety evaluation of RQ3025 in Sprague-Dawley rats. (A) The immunization scheme for Sprague-Dawley (SD) rats. Rats (*n* = 10 per group) were immunized three times on days 0, 14, and 28 through intramuscular injections with the following substances respectively: Saline (Control), low dose RQ3025 (10 μg), high dose RQ3025 (40 μg), low dose and high dose of empty LNP. Time points of vaccination, bleeding, and sample collection are indicated by arrows. (B, C) Hematological analysis in all five groups of rats. Percentages of lymphocyte subsets including CD3^+^, CD4^+,^ and CD8^+^, and expressions of cytokines IFN-γ, TNF-α, and IL-2 were monitored on days indicated. The black dashed line indicates the detection limit of the assay (IFN-γ, 9.40 pg/mL; TNF-α, 3.40 pg/mL; IL-2, 44.10 pg/mL). Any measurement below the detection limit was assigned a value of half the limit of detection for plotting and statistical purposes. Values are mean ± SEM. (D) Histopathological evaluations (H&E staining) in lungs from three groups of rats on day 31. Scale bar, 200 μm. Statistical analyses were performed using ANOVA and Tukey’s multiple comparison tests.

## Data Availability

Data supporting the findings in this study are included in the main article and associated files.

## Results

### Antigen design and characterization

RQ3025 is a bivalent broad-spectrum mRNA vaccine comprising two distinct mRNA molecules, namely RQ3013 and RQ3019, both based on the S protein sequences of SARS-CoV-2 variants. Both mRNAs encode a nearly full-length S protein excluding the eighteen cysteine-rich residues at the C-terminus. The S protein encoded by RQ3013 contains all mutations present in the B.1.1.7 variant, along with additional K417N, E484 K, and A701 V mutations found in the B.1.351 variant. Seven of these mutations also exist in the Omicron variant B.1.1.529 (Figure 1(A)). RQ3019 incorporates all mutations from the BA.2 variant, with additional L452R and F486 V mutations found in the BA.4/BA.5 variant (Figure 1(A)). Unlike the S2P design employed in BNT162b2 and mRNA-1273 vaccines [17,18], Immunogens encoded by RQ3013 and RQ3019 do not contain the two proline substitutions. Instead, the furin cleavage site ^682^RRAR^685^ is replaced by GSAS to prevent protease-mediated proteolysis. The native signal peptide and transmembrane domain are retained in both S proteins.

The mutation sites in RQ3025 cover all critical mutations found in 14 different variant strains including current VOIs such as BA.2.75, XBB.1.5, and VUMs such as XBB.1.16 and CH.1.1 (Figure 1(B)). Well-established lipid nanoparticles (LNPs) are used for mRNA encapsulation. The mRNA encapsulation efficiency (EE) for all three LNP vaccine candidates exceeded 90%, with an average diameter of 70 nm (Figure S1A). Moreover, all LNPs exhibited a consistent polydispersity index (PDI) of approximately 0.1, indicative of a uniform particle size distribution (Figure S1A). Furthermore, LNPs encapsulating mRNA consistently exhibited a negative surface charge (Figure S1B). The immunoreactivity of antigens derived from RQ3025 was assessed using multiple antibodies targeting distinct domains of the SARS-CoV-2 spike protein in western blots. The antibodies exhibited discriminative recognition of S proteins derived from RQ3013 or RQ3019 transfected HEK293 T cells on western blots, demonstrating mutation-directed immune specificity of the S proteins (Figure 1(C)). Cell lysates from HEK293 T transfected with RQ3013, RQ3019, or RQ3025 displayed bands similar to the purified proteins derived from RQ3013 and RQ3019 in the blots. These bands were consistent across all tested antibodies and conditions, with or without DTT. This confirms the integrity of the translated antigens from the mRNAs (Figure 1(C)). Cellular localization of the translated antigens from RQ3025 mRNA was assessed by immunofluorescence using antibodies recognizing either the receptor binding domain (RBD) of the spike protein from SARS (Figure 1(D), left panel) or SARS-CoV-2 (Omicron)-RBD (Figure 1(D), right panel). The SARS-RBD antibody exclusively detected signals on the surface of RQ3013 transfected cells. In contrast, the SARS-CoV-2 (Omicron)-RBD antibody detected signals in both RQ3013 and RQ3019 transfected cells. This difference reflects the antibody evasion ability of the Omicron variants. Both antibodies displayed signals localized to the membrane in RQ3025-transfected cells (Figure 1(D)). These results demonstrate the correct expressions and the immune characterizations of the antigens derived from RQ3025 *in vitro*.

### Immunogenicity of RQ3025 in BALB/c mice

To assess the immunogenicity of RQ3025 in BALB/c mice, two groups (*n* = 24) were immunized intramuscularly with either 2 μg or 5 μg of RQ3013, RQ3019, or RQ3025 on day 0. Control mice (*n* = 24) received saline (Figure 2(A)). All groups received booster injections on day 21. No local inflammation or other adverse effects were observed throughout the experiment. Sera were collected on day 28 to measure the immunobinding of IgGs to RBD, S and S2 by enzyme-linked immunosorbent assay (ELISA). RQ3013, RQ3019, and RQ3025 induced equally high titers of IgG binding to RBD, S, and S2 from the wild-type, B.1.351, and BA.5 variants (Figure 2(B)). There was no difference between the low-dose (2 μg) and the high-dose (5 μg) groups immunized with the same vaccine (Figure 2(B)). Next, we performed the lentiviral pseudovirus neutralization assay to evaluate the levels of neutralizing antibodies induced by RQ3013, RQ3019 or RQ3025 on day 28. RQ3013 elicited high titers of neutralizing antibodies against the B.1.351 variant in BALB/c mice. For several Omicron subvariants, such as BA.1, BA.2, BA.2.3.20, BA.2.38, BA.2.75, and BA.4/BA.5, RQ3013 also induced an effective amount of neutralizing antibodies. However, the neutralizing capacity of RQ3013 against BF.7, BL.1, BQ.1.1, XBB.1, XBB.1.5, CH.1.1 and EG.5.1 subvariants was significantly reduced (Figure 2(C)). In contrast, RQ3019 induced high titers of neutralizing antibodies against various Omicron subvariants, albeit with subdued neutralization ability against B.1.351 (Figure 2(C)). Immunization with the bivalent vaccine RQ3025 in BALB/c mice generated high titers of neutralizing antibodies against B.1.351 and all tested Omicron subvariants, e.g. geometric mean titer (GMT) = 4846 for XBB.1.5 and GMT = 1918 for EG.5.1 (Figure 2(C)). Sera were collected from the RQ3025-immunized mice on day 42 for assessing the neutralizing titer against two live virus strains (B.1.351 and XBB.1.5) by Focus Forming Assay (FFA). In mice that received a 2-µg dose, the FRNT_50_ GMT of neutralizing antibodies against B.1.351 and XBB.1.5 were 3286 and 1064, respectively. A slightly higher neutralizing titer against B.1.351, but not XBB.1.5, was observed in mice that received a 5-µg dose (Figure 2(D)). These results demonstrate excellent immunogenicity and broad neutralizing capacity of the bivalent mRNA vaccine RQ3025. Furthermore, long-term monitoring of the neutralizing antibody levels targeting B.1.351 and BA.4/BA.5 in BALB/c mice immunized with RQ3025 revealed persistent high titers of neutralizing antibodies against B.1.351 and BA.4/BA.5 in the mice 24 weeks post-immunization (Figure S2). Cell-mediated immunity was evaluated in the BALB/c mice immunized with RQ3025 or RQ3013 by using an IFN-γ ELISpot assay on isolated spleen cells restimulated with a pool of peptides derived from the S1 or S2 subunit. Both vaccines at low and high doses led to a strong cellular immune response in the spleen 3 months after the last immunization (Figure 2(E)). Intracellular cytokine staining assays measuring expressions of IFN-γ, TNF-α, IL-2, IL-4 or IL-5 in S1 or S2 peptides stimulated spleen cells showed polyfunctional CD4^+^ and CD8^+^ T cell responses were induced by RQ3025 immunization, with a higher CD8^+^ T cell response for S1 than S2 (Figure 2(F)). The T cell response induced by RQ3025 was comparable to that of RQ3013, indicating that the difference between RQ3025 and RQ3013 had little impact on cellular immunity (Figure 2(E,F)). It has been reported that vaccine-associated enhanced respiratory disease (VAERD) was associated with a Th2-dominant immune response in SARS-CoV-2 infected hamsters [19]. Therefore, the cytokines secreted by spleen cells upon restimulation with S1 and S2 peptide pools were evaluated to determine the T cell response type in RQ3025 immunized mice. The ratio of IFN-γ (Th1-associated cytokine) concentration to IL-4, IL-5, and IL-10 (Th2-associated cytokines) was 10–100 in S1 peptides stimulated splenocytes and approximately 10 in S2 peptides stimulated splenocytes, suggesting RQ3025 induced a Th1-dominant cell response (Figure 2(G)).

### Immunogenicity of RQ3025 in K18-hACE2 transgenic mice

We next assessed the immunogenicity and efficacy of RQ3013, RQ3019 and RQ3025 in the K18-hACE2 transgenic mouse strain which is susceptible to SARS-CoV-2 and suffers from lung injuries that mimic severe SARS-CoV-2 infection in humans [20–22]. Each mouse received two doses of either 2 or 5 μg injected on day 0 and day 21 (Figure 3(A)). Both the 2- and 5-μg doses of all three vaccines elicited high titers of RBD- and S-specific IgG antibodies in the sera of K18-hACE2 mice at 7 days post-boost (Figure 3(B)). Neutralizing antibodies in the sera were assessed by cell-based pseudovirus entry assay using viruses pseudotyped with the spike proteins of variant strains B.1.351, BA.4/5, BA.2.3.20, BQ.1.1, XBB.1, XBB.1.5, and CH.1.1. In consistence with the observations in BALB/c mice, RQ3025 elicited high levels of neutralizing antibodies against both the B.1.351 variant and the Omicron subvariants in comparison with RQ3013 and RQ3019 (Figure 3(C)). Administration of two doses of RQ3025 (2 µg) induced neutralizing antibody titers of GMT 1878, 4246, 2792, 18,905, 2984, 681 and 1740 against B.1.351, BA.4/BA.5, BA.2.3.20, BQ.1.1, XBB.1, XBB.1.5, and CH.1.1, respectively (Figure 3(C)). Spleen cells were isolated from the K18-hACE2 mice immunized with RQ3025 two months after the second injection and stimulated with a pool of S1 or S2 peptides. Cell-mediated immune response was triggered, which was evident from the elevated percentages of IFN-γ, TNF-α and IL-2 expressing CD4^+^ and CD8^+^ T cells (Figure 3(D)). To assess the durability of immunity, serum samples were collected at 40 weeks post-initial immunization, and the presence of neutralizing antibodies was determined via a pseudovirus entry assay. The data revealed a gradual decline in the levels of neutralizing antibodies against both the B.1.351 variant and the Omicron subvariants from 4 weeks to 36 weeks, with these levels stabilizing by the 40th week (Figure S3A). In terms of the cellular immune response, splenocytes were harvested 40 weeks after the second immunization and subsequently stimulated with the same pool of peptides. A consistent immune response was observed when compared to the response at 2 months (Figure S3B).

### Protection against SARS-CoV-2 variants in RQ3025 vaccinated K18-hACE2 transgenic mice

To measure the protective effect of RQ3025, K18-hACE2 mice vaccinated with RQ3025 or saline were challenged intranasally with B.1.351 (1 × 10^3^ PFU) 42 days post-boost or BA.5 (0.7 × 10^5^ PFU) 23 days post-boost (Figure 4(A)). High live virus neutralizing titers against both B.1.351 and BA.5 variants were detected at 0 and 4 days post-infection (4 dpi) in sera from mice vaccinated with RQ3025. In addition, neutralizing antibody titers against B.1.351 in RQ3025-vaccinated mice were higher at 5 dpi than at 0 dpi (Figure 4(B)). Mice infected with B.1.351 suffered more than a 10% body weight loss from 2 to 4 dpi, whereas those vaccinated with RQ3025 maintained their body weights (Figure 4(C)). An significant reduction in viral load in the lung, trachea, and brain was detected in RQ3025-vaccinated mice at 4 dpi (Figure 4(D)). Lung tissues at 4 dpi were sectioned and examined by hematoxylin and eosin (H&E) staining to evaluate lung injuries induced by B.1.351 infection. Control mice infected with B.1.351 exhibited severe lung injuries as evidenced by alveolar collapse, thickened alveolar septa, and extensive infiltration of inflammatory cells at 4 dpi, whereas mice vaccinated with RQ3025 showed mild to moderate inflammatory cell infiltration, and alveolar structures were maintained (Figure 4(E) and Figure S5A). In mice challenged with BA.5 infection high titers of live virus neutralizing antibodies against both B.1.351 and BA.5 variants were also detected at 0 and 5 dpi in mice vaccinated with RQ3025 and neutralizing antibody titers against BA.5 in RQ3025-vaccinated mice were higher at 5 dpi than at 0 dpi (Figure 4(F)). Control mice infected with BA.5 experienced over a 20% body weight loss from 1 to 5 dpi, whereas in mice vaccinated with RQ3025 (2 and 5 µg) body weight loss was averted from 3 dpi (Figure 4(G)). RQ3025 vaccination significantly reduced the viral load in the lung, trachea and brain at 5 dpi (Figure 4(H)). Lung tissues in RQ3025-vaccinated mice were protected from BA.5-induced injuries (Figure 4(I) and Figure S5B). Together, RQ3025 induced humoral and cellular immune responses, providing protection against B.1.351 and BA.5 infection in K18-hACE2 mice.

### Immunogenicity and protection of RQ3025 in hamsters

The Syrian hamster, expressing the ACE2 receptor, is highly susceptible to SARS-CoV-2 and develops pneumonia similar to that in COVID-19 patients, making it a suitable model for vaccine evaluation [23]. Seven groups of hamsters were vaccinated on day 0 and day 21 with saline or the three vaccines (RQ3013, RQ3019 and RQ3025) at two doses (5 or 25 μg) followed by immunogenicity evaluation and viral challenge (Figure 5(A)). High titers of antibodies binding to the RBD, S (B.1.351 and BA.5) and S2 subunit were detected in sera collected from RQ3025-immunized hamsters on day 28 (Figure 5(B)). A pseudovirus neutralization assay with a panel of variant strains was used to test the range of neutralizing antibodies elicited by the three vaccines. RQ3025 elicited neutralizing antibodies against all variants while neutralizing antibodies induced by RQ3013 and RQ3019 were effective only against either B.1.351 or Omicron subvariants (Figure 5(C)). Immunization with RQ3025 at a 25 μg dose elicited serum neutralizing antibody titers averaging 3246, 2941, 900, 2181, and 1120 against B.1.351, BA.4/BA.5, BA.2.3.20, BQ.1.1, and XBB.1 strains, respectively (Figure 5(C)). Neutralizing titers against live virus B.1.351 and BA.5 were measured using a live virus microneutralization assay which also demonstrated the effectiveness of RQ3025 against both strains in comparison to the limited neutralizing abilities of RQ3013 and RQ3019 (Figure 5(D)). Twenty-two days following the second vaccination, hamsters were challenged with the BA.5 variant (0.7 × 10^5^ PFU). Neutralizing antibodies against both B.1.351 and BA.5 live viruses in sera were detected at 0 and 5 dpi. A significant increase in neutralizing antibody against BA.5 was induced at 5 dpi and was further enhanced in hamsters vaccinated with 25 µg RQ3025 (Figure 5(E)). Body weights of control hamsters slightly decreased at 5 dpi, whereas hamsters vaccinated with 25 µg RQ3025 exhibited an increase in body weight from 3 to 5 dpi. The 5-µg dose of RQ3025 immunization did not show an effect on body weight compared to the control (Figure 5(F)). The viral load in the lungs of hamsters immunized with both doses of RQ3025 was significantly lower than that in the control at 5 dpi (Figure 5(G)). Hamsters in the control group suffered lung injuries with thickened alveolar septa and a large number of inflammatory cells infiltration at 5 dpi while hamsters immunized with RQ3025 (5 and 25 µg) showed mild to moderate inflammatory cell infiltration and mild alveolar epithelial hyperplasia (Figure 5(H) and Figure S5C). Together, these results provided evidence that RQ3025 vaccination elicits protection against SARS-CoV-2 variants in Syrian hamster.

### Immunogenicity of RQ3025 as a third booster in BALB/c mice

We next investigated the feasibility of mRNA vaccines as a booster following inoculation with inactivated vaccines. Mice immunized with two doses of inactivated vaccines on day 0 and day 21 received a third booster with an inactivated vaccine or mRNA vaccines RQ3013 or RQ3025 on day 77 (56 days after the second dose of the inactivated vaccine) (Figure 6(A)). Sera were collected on day 105 for assessment of antibodies. A booster with either RQ3013 or RQ3025 elicited higher titers of RBD-, S-, and S2-specific IgGs than a booster with the inactivated vaccine (Figure 6(B)). A booster with RQ3025 was the most effective in inducing specific antibodies binding to the BA.5 S protein (Figure 6(B)). RQ3025 effectively boosted the neutralizing antibodies against both B.1.351 and Omicron variants (BA.4/BA.5, BQ.1.1 and XBB.1) whereas the third boost with the inactivated vaccine or RQ3013 failed to enhance the neutralizing capability against BQ.1.1 and XBB.1 (Figure 6(C)). Splenocytes were isolated on day 171 (3 months after the third boost) for the assessment of cell-mediated immunity. RQ3025 induced higher cellular immune response than the inactivated vaccine, as measured by the amount of IFN-γ and IL-2 secreting spleen cells (Figure 6(D)). Furthermore, mice immunized with RQ3025 as the third booster showed a significantly increased percentage of CD4^+^ and CD8^+^ T cells expressing IFN-γ, IL2 and TNF-α, indicating a higher polyfunctional CD4^+^ and CD8^+^ T cell response compared to that with the inactivated vaccine (Figure 6(E), with the gating strategy for FACS shown in Figure S4). These results suggested that using RQ3025 as a booster in mice previously immunized with inactivated vaccines can provide protection against a range of SARS-CoV-2 variants by enhancing both humoral and cell-mediated immunity.

### Safety and immunogenicity of RQ3025 in Sprague–Dawley rats

The safety of RQ3025 was evaluated in Sprague–Dawley rats, a strain widely acknowledged as suitable for toxicological research on drugs that have been or are intended to be used in humans [24]. Fifty rats were equally divided into five groups. Each group, on day 0, received intramuscularly one dose of either saline, 10 or 40 μg of RQ3025, or empty LNPs (0.22 mg or 0.86 mg of total lipid) (Figure 7(A)). All groups received booster injections on days 14 and 28. Neither fever nor weight loss was observed in all animals (Figure S6). Blood samples were collected at various time points for hematological and biochemical analysis. No significant changes were detected in hematological indices or in the percentages of CD3^+^, CD4^+^, and CD8^+^ T cells between the groups (Figure S7 and Figure 7(B)). The secretions of Th1 cytokines (IFN-γ, TNF-α, and IL-2) and Th2 cytokines (IL-6) were not changed by injections of RQ3025 or empty LNP (Figure 7(C) and Figure S8). Rats injected with saline or 40 µg of RQ3025 were euthanized on day 31. Lungs, brains, hearts, livers, spleens, and kidneys were harvested and sectioned for H&E staining for histopathological analysis. Vaccine-associated immunopathologic changes were not observed in any of the sections from these tissues in all animals (Figure 7(D) and Figure S9), suggesting that the administration of RQ3025 at a high dose (40 µg) was safe in rats.

The immunogenicity of RQ3025 in Sprague–Dawley rats was also assessed. Rats immunized with 10 μg and 40 μg doses of RQ3025 induced high titers of IgGs binding to RBD (wild-type), S (wild-type, B.1.351 and BA.5), and S2 (Figure S10A). High titers of neutralizing capacity against B.1.351 and a range of Omicron subvariants (BA.2.3.20, BA.4/BA.5, BQ.1.1, XBB.1, XBB.1.5 and CH.1.1) in the sera of vaccinated rats were demonstrated by a pseudovirus virus neutralization assay (Figure S10B). The seroconversion rate was 100% for B.1.351 and all the tested Omicron subvariants (Figure S10B). These data demonstrate the safety of RQ3025 and its broad-spectrum cross-neutralization activity in rats, supporting its candidacy for clinical development.

## Discussion

Amid the COVID-19 pandemic, mRNA vaccine technology demonstrated a great advantage in quick response, contributing largely to the establishment of population-level immunity against SARS-CoV-2. The World Health Organization declared an end to COVID-19 as a global health emergency on 5 May 2023. However, risks remain as the virus continue to evolve and new variants emerge. The development of vaccines that provide broad-spectrum protection is critical to prevent surges in cases and hospitalizations caused by new variants. Here, we present comprehensive assessments of a novel bivalent mRNA vaccine, RQ3025, evaluating its immunogenicity, efficacy, and safety in animal models, providing compelling evidence for its further exploration in clinical trials.

The mRNA vaccines mRNA-1273 and BNT162b2 developed soon after the onset of the COVID-19 pandemic demonstrated over 90% protection efficacy during the initial stages of the pandemic [25]. However, SARS-CoV-2 variants such as Delta and Omicron with immune escape capacity through mutations in the spike protein emerged quickly and significantly reduced the protective efficacy of the vaccines [4,7,10]. It is difficult to predict mutations that can lead to high infectivity and strong immune escape capacity in emerging variants. The vaccine development strategy based on a single strain struggles to keep pace with the rapid evolution of the virus. Various evolutionary paths of SARS-CoV-2 have been identified, and a universal antigen designed based on a comprehensive analysis of evolutionary mutation sites in the spike protein sequences of SARS-CoV-2 variants could effectively induce broad neutralizing antibodies [26,27]. Currently, bivalent mRNA vaccines are widely used to maintain protection efficacy against rapidly emerging SARS-CoV-2 variants, although results were subtle when used as a booster dose [28]. The newly developed bivalent mRNA vaccine RQ3025 presented in this study comprises two mRNA sequences (RQ3013 and RQ3019) encoding chimeric S proteins incorporating key mutation sites found in Alpha, Beta, and Omicron BA.2/BA.4/BA.5 variants of SARS-CoV-2. Most of these mutations exhibited evolutionary conservation and were harbored by numerous recent variants, including BQ.1.1, XBB.1.5, CH.1.1 and XBB.1.16. Our results in various animal models demonstrated that RQ3025 induced high levels of broad neutralizing antibodies against a range of variants including B.1.351, BA.2, BA.2.3.20, BA.2.38, BA.2.75, BA.4, BA.5 BF.7, BQ.1.1, XBB.1, XBB.1.5, XBB.1.16 and CH.1.1, in contrast to the limited neutralizing capacity shown by the monovalent vaccine RQ3013 and RQ3019. With seroconversion rates of 100% for most of these variants, RQ3025 appears to induce relatively unbiased cross-neutralizing antibody responses, attributed to its broad coverage of mutations on the spike protein. Two doses of RQ3025 effectively suppressed the replication of the Omicron variant BA.5 and ameliorated lung injury caused by viral infection in both mice and hamsters. Robust T-cell immune responses persisted for three and two months after the second immunization with RQ3025 in BALB/c and K18-hACE2 mice, respectively, underscoring the vaccine's durable protective efficacy.

In developing vaccines against SARS-CoV-2, concerns about VAERD arose, although it has not been reported in humans receiving current vaccines [1,2]. It has been reported that VAERD in SARS-CoV-2-infected Syrian hamsters immunized with a vaccine consisting of misfolded spike protein and aluminum-containing adjuvants is associated with a Th2-biased immune response^1^. In our study, a Th1-biased immune response was evident after stimulation with S1 or S2 peptide pools in splenocytes isolated from RQ3025-vaccinated mice, indicating that VAERD is unlikely to develop in RQ3025-vaccinated BALB/c mice.

Currently, the vaccination rate with inactivated COVID-19 vaccines has surpassed 90% in China [29], and a portion of the global population has also received inactivated COVID-19 vaccines. In this study, we evaluated the immunogenicity of different vaccines as booster doses given to BALB/c mice previously immunized with two doses of an inactivated COVID-19 vaccine. The results revealed that a booster dose with RQ3025 generated broader protection with high-titer neutralizing antibodies against B.1.351 and Omicron subvariants (BA.4/BA.5, XBB.1, and BQ.1.1) and induced strong cell-mediated immunity, as shown by a specific T cell immune response against the S protein, demonstrating significant advantages over the inactivated vaccine and monovalent mRNA vaccine RQ3013.

To date, hundreds of SARS-CoV-2 variants have been documented from human patients and the virus continue to evole rapidly. Recently, accelerated evolution of SARS-CoV-2 has been discovered in animal hosts [30]. It is essential to continuously monitor emerging variants and adjust strategies for vaccine development and administration accordingly as new variants may cause serious consequences for human society in the future. The preclinical evaluation of the bivalent mRNA vaccine RQ3025 in this study provides evidences of its excellent broad protective capacity against a range of current variants and supports further exploration of its protective efficacy against current and future variant strains in human.

## References

[1] Polack FP, Thomas SJ, Kitchin N, et al. Safety and efficacy of the BNT162b2 mRNA COVID-19 vaccine. N Engl J Med. 2020;383(27):2603–2615. doi:10.1056/NEJMoa2034577 33301246 PMC7745181

[2] Baden LR, El Sahly HM, Essink B, et al. Efficacy and safety of the mRNA-1273 SARS-CoV-2 vaccine. N Engl J Med. 2021;384(5):403–416. doi:10.1056/NEJMoa2035389 33378609 PMC7787219

[3] Faria NR, Mellan TA, Whittaker C, et al. Genomics and epidemiology of the P.1 SARS-CoV-2 lineage in Manaus, Brazil. Science. 2021;372(6544):815–821. 33853970 10.1126/science.abh2644 PMC8139423

[4] Planas D, Veyer D, Baidaliuk A, et al. Reduced sensitivity of SARS-CoV-2 variant delta to antibody neutralization. Nature. 2021;596(7871):276–280. doi:10.1038/s41586-021-03777-9 34237773

[5] Tegally H, Wilkinson E, Giovanetti M, et al. Detection of a SARS-CoV-2 variant of concern in South Africa. Nature. 2021;592(7854):438–443. doi:10.1038/s41586-021-03402-9 33690265

[6] Volz E, Mishra S, Chand M, et al. Assessing transmissibility of SARS-CoV-2 lineage B.1.1.7 in England. Nature. 2021;593(7858):266–269. doi:10.1038/s41586-021-03470-x 33767447

[7] Collie S, Champion J, Moultrie H, et al. Effectiveness of BNT162b2 vaccine against Omicron variant in South Africa. N Engl J Med. 2022;386(5):494–496. doi:10.1056/NEJMc2119270 34965358 PMC8757569

[8] Islam MR, Shahriar M, Bhuiyan MA. The latest Omicron BA.4 and BA.5 lineages are frowning toward COVID-19 preventive measures: A threat to global public health. Health Sci Rep. 2022;5(6):e884. doi:10.1002/hsr2.884 36254237 PMC9561426

[9] Lu L, Mok BWY, Chen LL, et al. Neutralization of severe acute respiratory syndrome coronavirus 2 omicron variant by sera from BNT162b2 or CoronaVac vaccine recipients. Clin Infect Dis. 2022;75(1):e822–e826. doi:10.1093/cid/ciab1041 34915551 PMC8754807

[10] Cele S, Jackson L, Khoury DS, et al. Omicron extensively but incompletely escapes Pfizer BNT162b2 neutralization. Nature. 2022;602(7898):654–656. doi:10.1038/s41586-021-04387-1 35016196 PMC8866126

[11] Dejnirattisai W, Huo J, Zhou D, et al. SARS-CoV-2 Omicron-B.1.1.529 leads to widespread escape from neutralizing antibody responses. Cell. 2022;185(3):467–484.e15. doi:10.1016/j.cell.2021.12.046 35081335 PMC8723827

[12] Liu L, Iketani S, Guo Y, et al. Striking antibody evasion manifested by the Omicron variant of SARS-CoV-2. Nature. 2022;602(7898):676–681. doi:10.1038/s41586-021-04388-0 35016198

[13] Wang Q, Bowen A, Valdez R, et al. Antibody response to omicron BA.4-BA.5 bivalent booster. N Engl J Med. 2023;388(6):567–569. doi:10.1056/NEJMc2213907 36630643 PMC9847504

[14] Collier AY, Miller J, Hachmann NP, et al. Immunogenicity of BA.5 bivalent mRNA vaccine boosters. N Engl J Med. 2023;388(6):565–567. doi:10.1056/NEJMc2213948 36630611 PMC9847505

[15] Lu J, Lu G, Tan S, et al. A COVID-19 mRNA vaccine encoding SARS-CoV-2 virus-like particles induces a strong antiviral-like immune response in mice. Cell Res. 2020;30(10):936–939. doi:10.1038/s41422-020-00392-7 32801356 PMC7429369

[16] Tan S, Hu X, Li Y, et al. Preclinical evaluation of RQ3013, a broad-spectrum mRNA vaccine against SARS-CoV-2 variants. Sci Bull (Beijing). 2023;68(2):3192–3206. doi:10.1016/j.scib.2023.11.024 37993332

[17] Walsh EE, Frenck RW, Falsey J, et al. Safety and immunogenicity of Two RNA-based COVID-19 vaccine candidates. N Engl J Med. 2020;383(25):2439–2450. doi:10.1056/NEJMoa2027906 33053279 PMC7583697

[18] Corbett KS, Edwards DK, Leist SR, et al. SARS-CoV-2 mRNA vaccine design enabled by prototype pathogen preparedness. Nature. 2020;586(7830):567–571. doi:10.1038/s41586-020-2622-0 32756549 PMC7581537

[19] Ebenig A, Muraleedharan S, Kazmierski J, et al. Vaccine-associated enhanced respiratory pathology in COVID-19 hamsters after TH2-biased immunization. Cell Rep. 2022;40(7):111214. doi:10.1016/j.celrep.2022.111214 35952673 PMC9346010

[20] McCray PB Jr., Pewe L, Wohlford-Lenane C, et al. Lethal infection of K18-hACE2 mice infected with severe acute respiratory syndrome coronavirus. J Virol. 2007;81(2):813–821. doi:10.1128/JVI.02012-06 17079315 PMC1797474

[21] Radvak P, Kwon HJ, Kosikova M, et al. SARS-CoV-2 B.1.1.7 (alpha) and B.1.351 (beta) variants induce pathogenic patterns in K18-hACE2 transgenic mice distinct from early strains. Nat Commun. 2021;12(1):6559. doi:10.1038/s41467-021-26803-w 34772941 PMC8589842

[22] Winkler ES, Bailey AL, Kafai NM, et al. SARS-CoV-2 infection of human ACE2-transgenic mice causes severe lung inflammation and impaired function. Nat Immunol. 2020;21(11):1327–1335. doi:10.1038/s41590-020-0778-2 32839612 PMC7578095

[23] Munoz-Fontela C, Dowling WE, Funnell SGP, et al. Animal models for COVID-19. Nature. 2020;586(7830):509–515. doi:10.1038/s41586-020-2787-6 32967005 PMC8136862

[24] Cavagnaro JA. Preclinical safety evaluation of biotechnology-derived pharmaceuticals. Nat Rev Drug Discov. 2002;1(6):469–475. doi:10.1038/nrd822 12119749

[25] Thompson MG, Burgess JL, Naleway AL, et al. Prevention and attenuation of COVID-19 with the BNT162b2 and mRNA-1273 Vaccines. N Engl J Med. 2021;385(4):320–329. doi:10.1056/NEJMoa2107058 34192428 PMC8262622

[26] Markov PV, Ghafari M, Beer M, et al. The evolution of SARS-CoV-2. Nat Rev Microbiol. 2023;21(6):361–379. doi:10.1038/s41579-023-00878-2 37020110

[27] Zhao Y, Ni W, Liang S, et al. Vaccination with S_pan_, an antigen guided by SARS-CoV-2 S protein evolution, protects against challenge with viral variants in mice. Sci Transl Med. 2023;15(677):eabo3332. doi:10.1126/scitranslmed.abo3332 36599007

[28] Offit PA. Bivalent COVID-19 vaccines — A cautionary tale. N Engl J Med. 2023;388(6):481–483. doi:10.1056/NEJMp2215780 36630616

[29] Cai J, Deng X, Yang J, et al. Modeling transmission of SARS-CoV-2 Omicron in China. Nat Med. 2022;28(7):1468–1475. doi:10.1038/s41591-022-01855-7 35537471 PMC9307473

[30] McBride DS, Garushyants SK, Franks J, et al. Accelerated evolution of SARS-CoV-2 in free-ranging white-tailed deer. Nat Commun. 2023;14(1):5105. doi:10.1038/s41467-023-40706-y 37640694 PMC10462754

