# Supplementary material for: Effectiveness of a broad-spectrum bivalent mRNA vaccine against SARS-CoV-2 variants in preclinical studies
Source: Emerg Microbes Infect. 2024 Feb 20;13(1):2321994. doi: 10.1080/22221751.2024.2321994 (PMC10906132; doi:10.1080/22221751.2024.2321994)
Supplement: Supplementary_Information_sent [file TEMI_A_2321994_SM8554.docx]

**Supplementary Information**

**
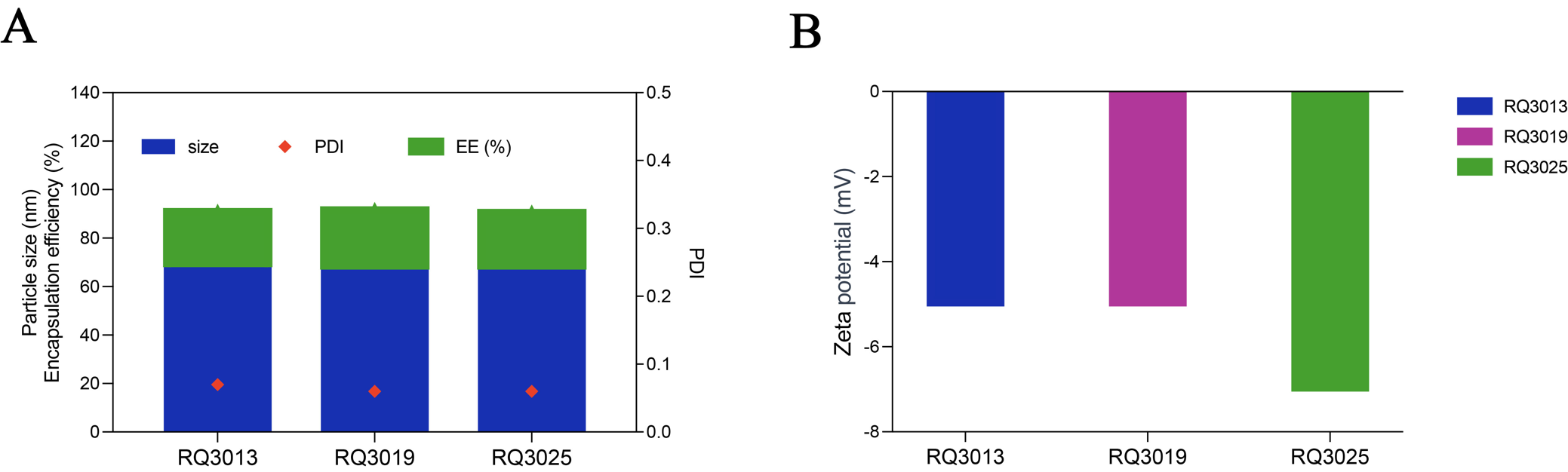
**

**Supplementary Figure 1.** Characterization of mRNA lipid nanoparticle formulations. (A) Analysis of particle size (represented by blue bar graph), polydispersity index (indicated by red diamond), encapsulation efficiency (depicted in the green bar graph), and (B) surface charge assessment of the LNPs, determined through zeta potential analysis.

**
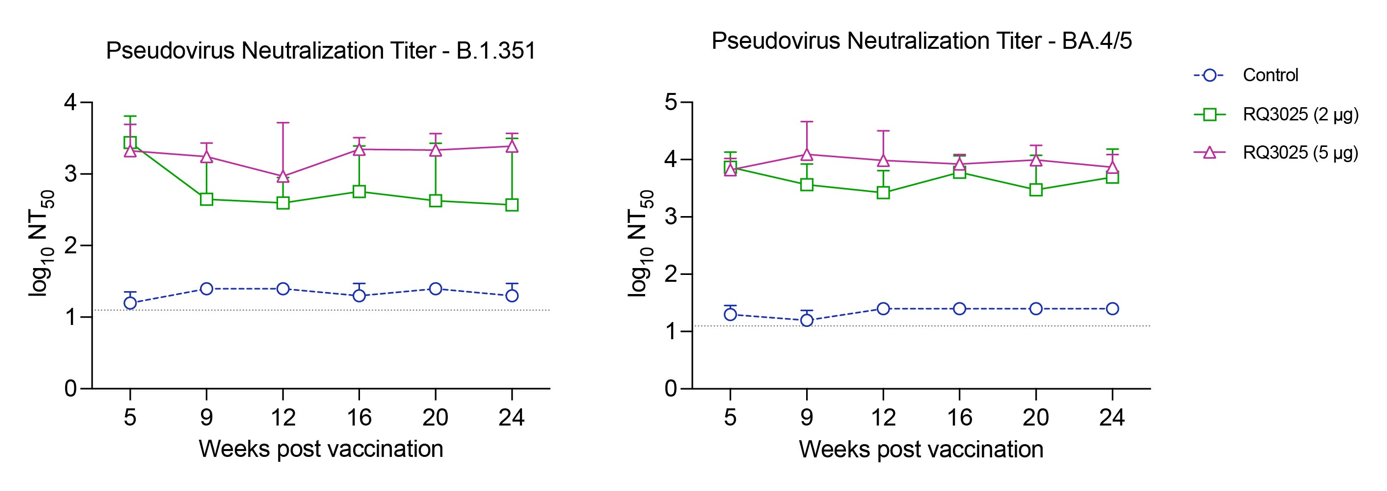
**

**Supplementary Figure 2.** Long-term monitoring of immunogenicity of RQ3025 in BALB/c mice. BALB/c mice were immunized two times on days 0 and 21 through intramuscular injections with low (2 μg) or high dose (5 μg) of RQ3025 or saline (Control). The antibody responses in sera from weeks 5 to 24 following the prime vaccination were analyzed by lentiviral luciferase-based pseudovirus assay. The black dashed line indicates the assay's detection limit (reciprocal titer of 12.5) (n = 6). Values are geometric mean ± SD.

**
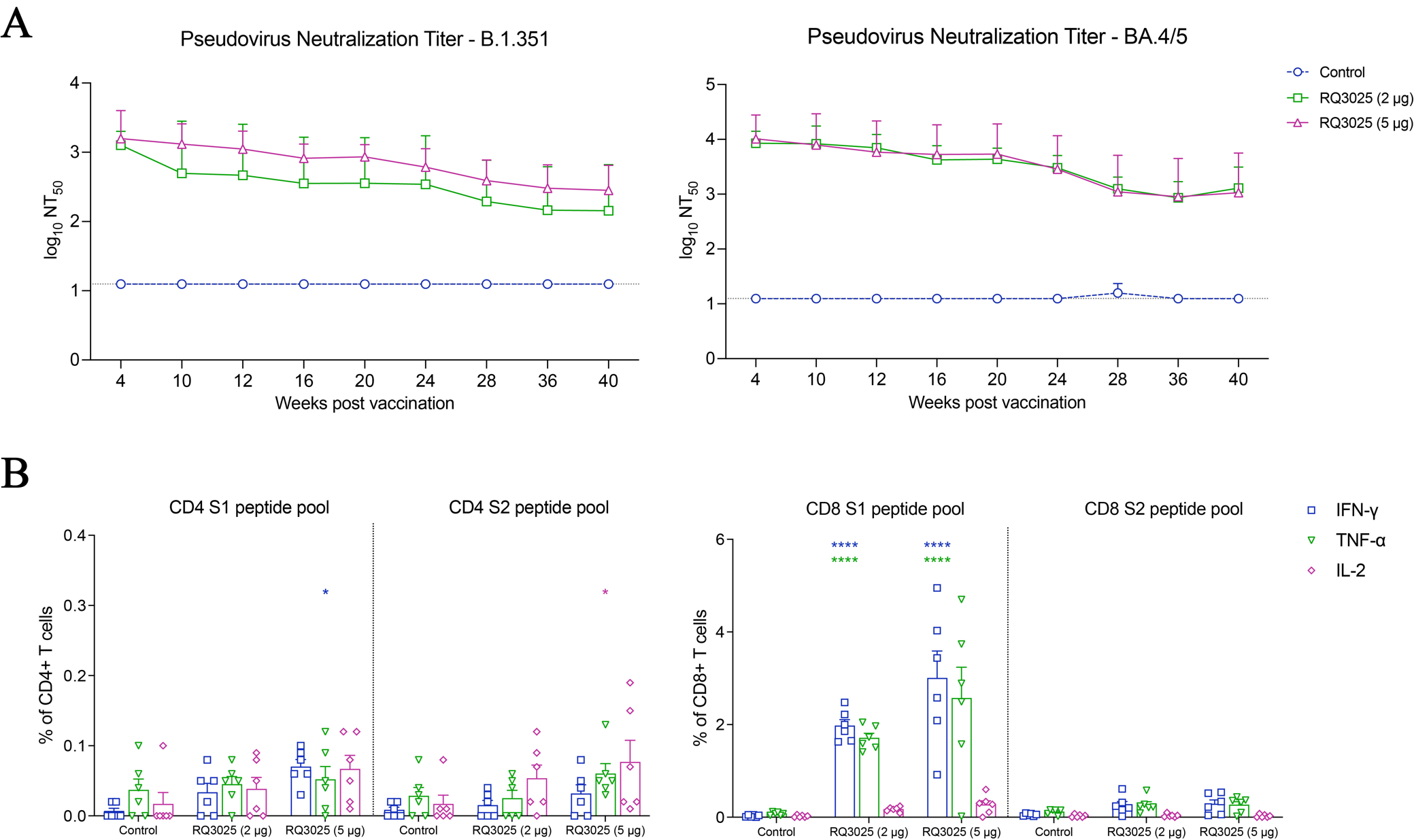
**

**Supplementary Figure 3.** Long-term monitoring of immunogenicity of RQ3025 in K18-hACE2 transgenic mice. Mice were subjected to two immunization events, administered on days 0 and 21 via intramuscular injections, involving either a low dosage (2 μg) or a high dosage (5 μg) of RQ3025 or a control group treated with saline solution. (A) Antibody responses in serum samples collected from weeks 4 to 40 post-initial vaccination were evaluated through a lentiviral luciferase-based pseudovirus assay. The assay's lower limit of detection is represented by the black dashed line (reciprocal titer of 12.5) (n = 6). Values are presented as geometric mean ± SD. (B) The percentages of CD4^+^ and CD8^+^ T cells expressing IFN-γ, IL-2, and TNF-α were assessed using intracellular cytokine staining and flow cytometry in splenocytes collected 40 weeks after the second immunization, following stimulation with a peptide pool derived from either the S1 or S2 regions of the SARS-CoV-2 Spike protein. Data are presented as mean ± SEM. The significance analysis was conducted in comparison with the control group. Statistical analyses were carried out by ANOVA and Tukey’s multiple comparison tests (***P* < 0.005; ****P* < 0.001; *****P* < 0.0001).


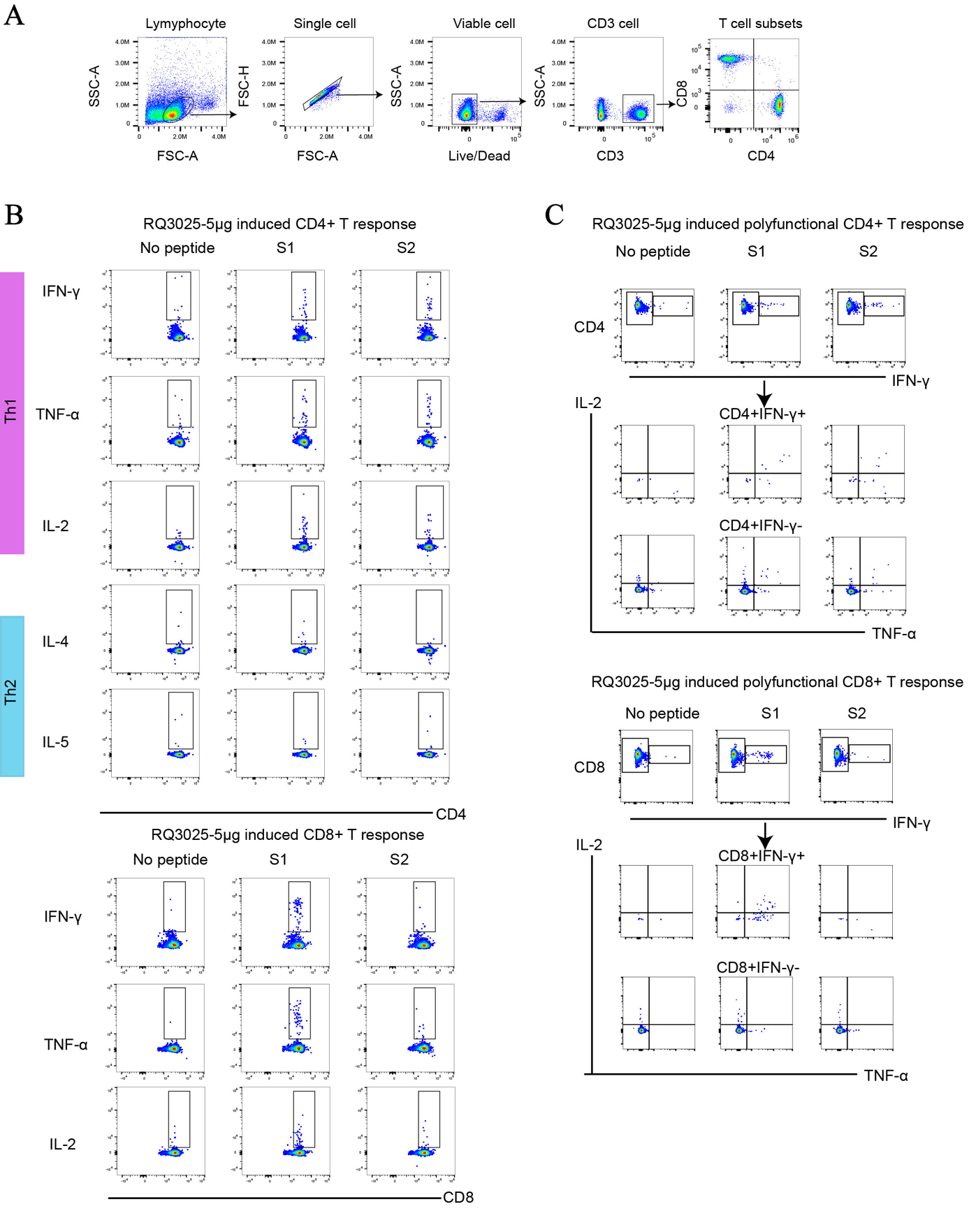


**Supplementary Figure 4.** Representative gating strategy for flow cytometry analysis of Spike-specific T cell response in BALB/c. (A) Gating strategy for T cell subsets. (B) Gating strategy for analyses of CD4^+^ and CD8^+^ T cells expressing IFN-γ, IL-2, TNF-α, IL-4 or IL-5. (C) Gating strategy for analysis of CD4^+^ T and CD8^+^ T cells expressing IFN-γ, IL-2 and/or TNF-α. FACS analysis on spleen cells from RQ3025 (5 μg) immunized mice are shown as examples.

**
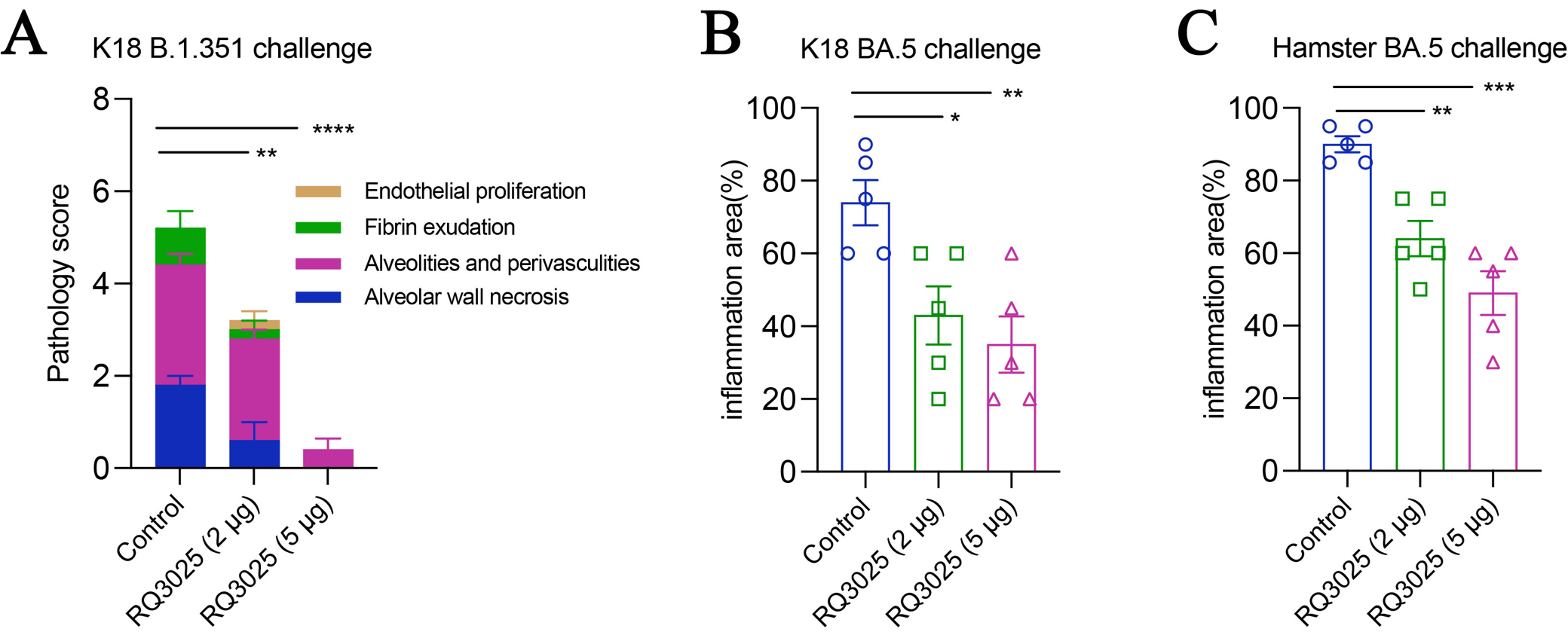
**

**Supplementary Figure 5.** Lung pathology score following challenge. Lung tissue from (a) K18 challenged with B.1.351. (b) K18 challenged with BA.5. (C) Golden hamster challenged with BA.5 was embedded in paraffin, sectioned, stained with hematoxylin and eosin, and was scored from 0 (no pathology) to 4 (severe pathology) or evaluated for inflammation area (%) by a blinded pathologist. Values are geometric mean ± SEM. Statistical analyses were carried out by ANOVA and Tukey’s multiple comparison tests (**P < 0.005; ****P < 0.0001).

**
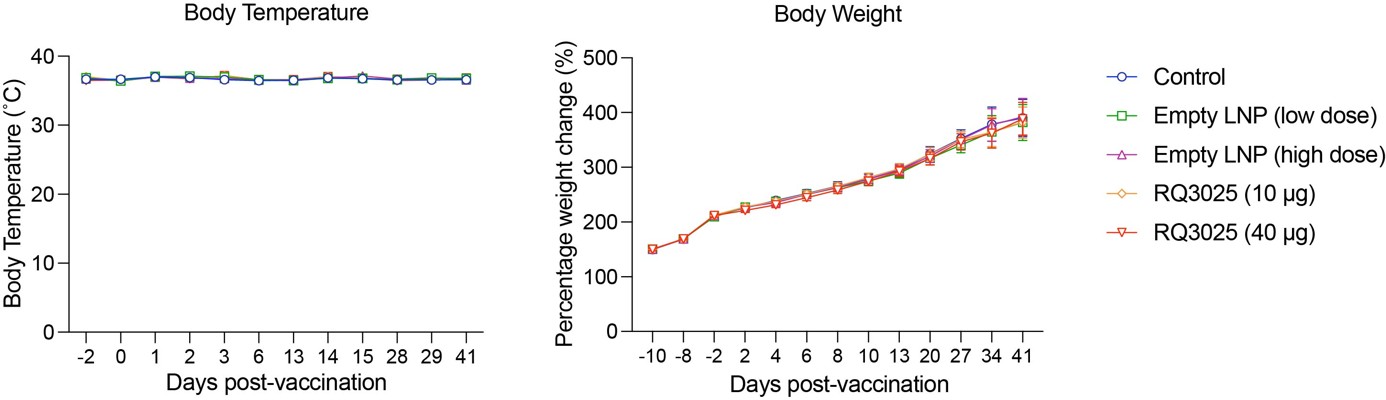
**

**Supplementary Figure 6.** Body weight and temperature changes in immunized Sprague-Dawley rats. Monitoring of body temperature (left) and body weight (right) in rats (n = 10 per group) vaccinated with saline (Control), low (10 μg) or high dose (40 μg) of RQ3025, low or high doses of empty LNP on days 0, 14, and 28 through intramuscular injections. Values are mean ± SEM.

**
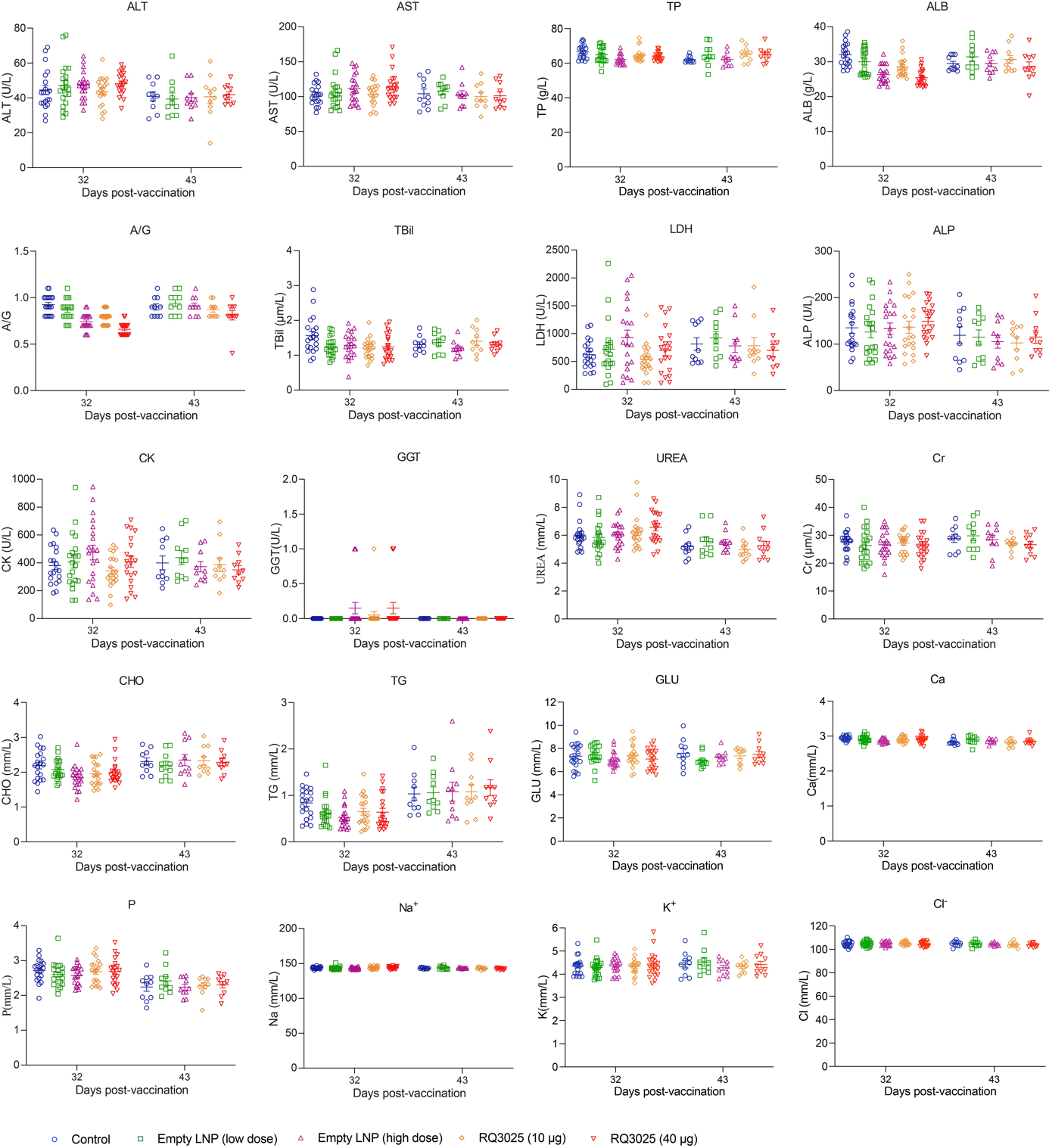
**

**Supplementary Figure 7.** Hematological indices in immunized Sprague-Dawley rats. The following hematological indices were measured in rats subjected to three injections (intramuscular, day 0, 14 and 28) of saline (Control), low dose (10 μg) or high dose (40 μg) of RQ3025, low or high doses of empty LNP on day 32 and 43 post-injection: ALT (Alanine aminotransferase), AST (Aspartate aminotransferase), ALP (Alkaline phosphatase), TBil (Total bilirubin), GGT (γ-glutamyltranspeptidase), TP (Total protein), Alb (Albumin), A/G (Albumin/globulin ratio), Glu (Glucose), UREA (Blood urea), Cre (Creatinine), CK (Creatine kinase), LDH (Lactate dehydrogenase), CHO (Total cholesterol), TG (Triglycerides), Ca (Calcium), P (Phosphorus), Na^+^ (Sodium ion), K^+^ (Potassium ion), Cl^-^ (Chloride ion). Values are mean ± SEM.

**
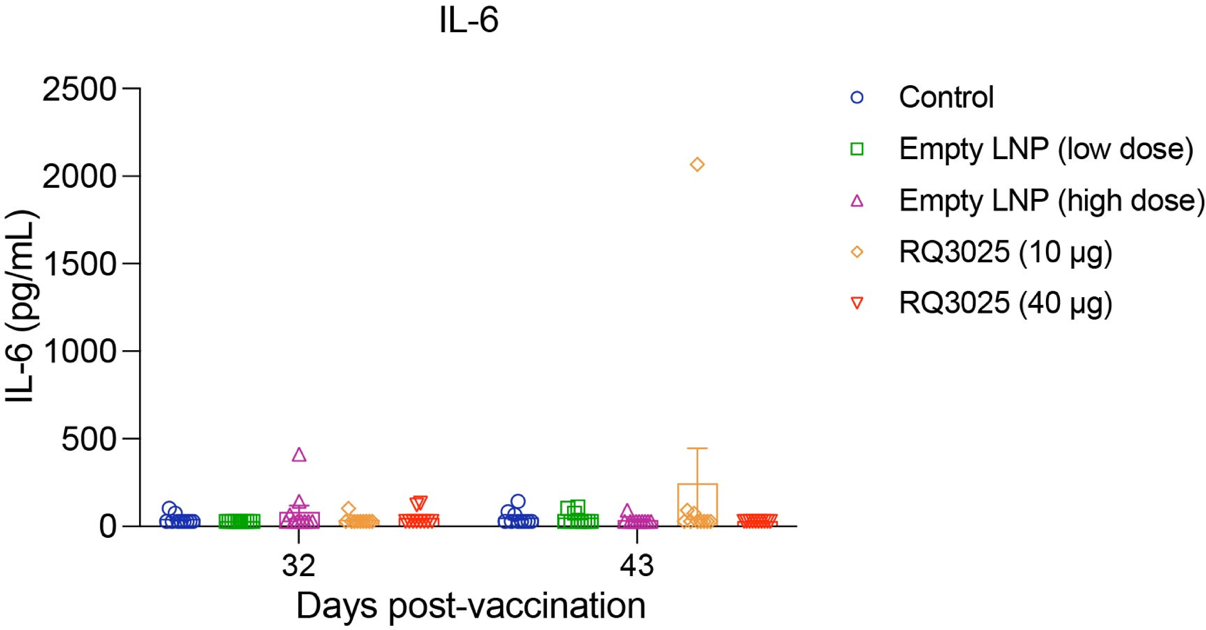
**

**Supplementary Figure 8.** IL-6 levels in immunized Sprague-Dawley rats. IL-6 levels were measured in rats subjected to three injections (intramuscular, day 0, 14 and 28) of saline (Control), low dose (10 μg) or high dose (40 μg) of RQ3025, low or high doses of empty LNP on day 32 and 43 post-injection. The black dashed line indicates the detection limit of the assay (IL-6, 14.52 pg/mL). Any measurement below the detection limit was assigned a value of half the limit of detection for plotting and statistical purposes. Values are mean ± SEM.

**
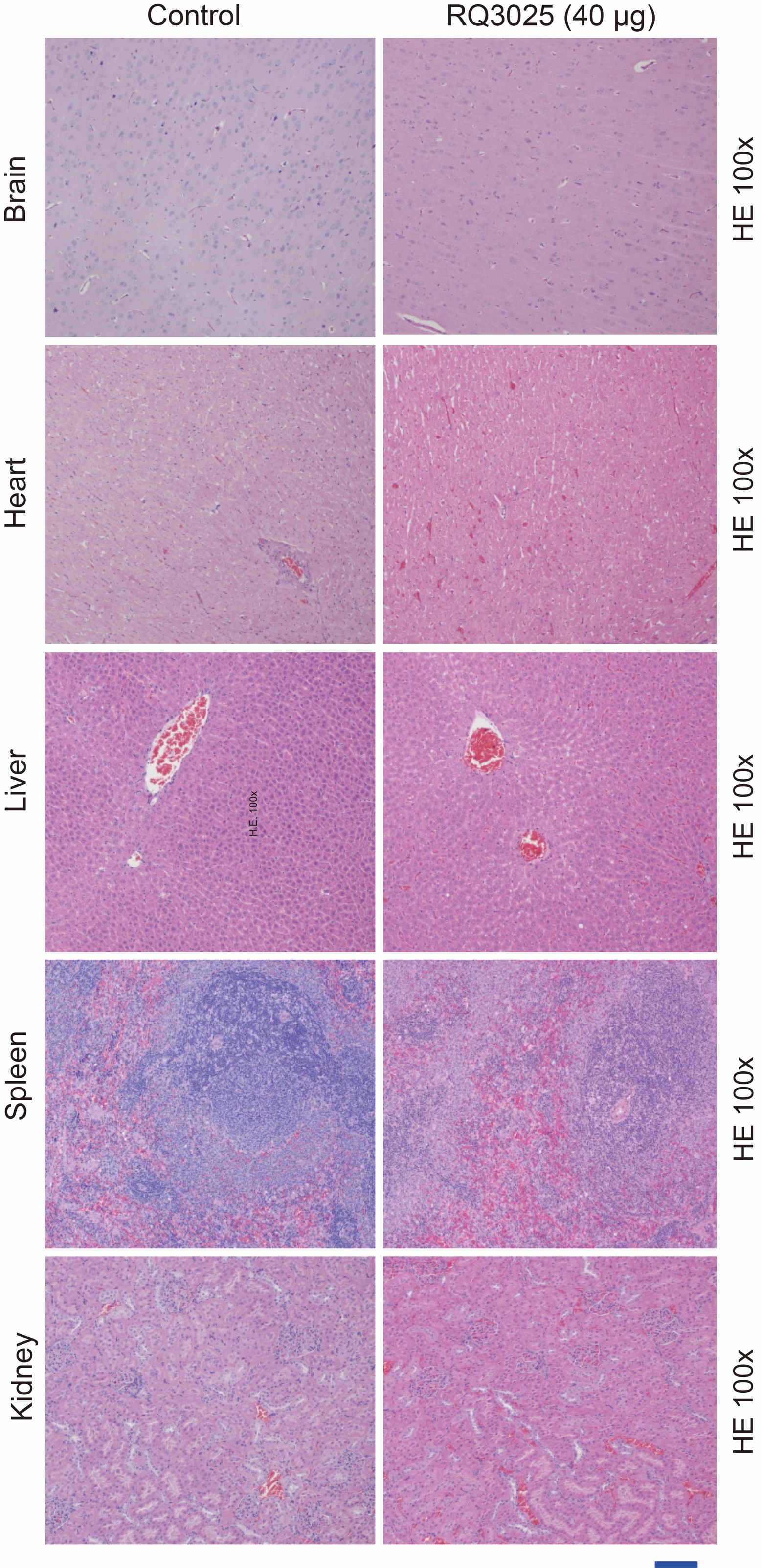
**

**Supplementary Figure 9.** Histopathological evaluations of RQ3025 safety in Sprague-Dawley rats. H&E staining on brain, heart, liver, spleen, and kidney tissues collected from rats inmmunized with control or high dose of RQ3025 (intramuscular injections on day 0, 14 and 28) on day 31. Scale bar, 200 μm.

**
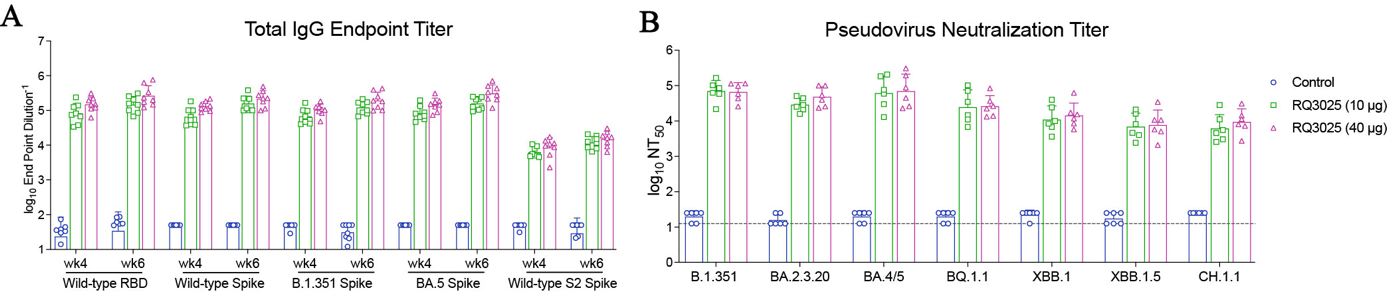
**

**Supplementary Figure 10.** Immunogenicity of RQ3025 in Sprague-Dawley rats. (A) In rats immunized (intramuscular injections on day 0, 14 and 28) with saline (Control), low dose (10 μg) or high dose (40 μg) of RQ3025, low or high doses of empty LNP, binding of IgGs to the wild-type RBD antigen, S proteins from the wild-type, B.1.351 and BA.5 viruses, and wild-type S2 subunit using sera collected on day 28 (before the third vaccination) and day 42 (14 days after the third vaccination) were measured by ELISA. (B) Neutralizing antibody titers in sera from day 42, analyzed by the lentiviral luciferase-based pseudovirus assay. The black dashed line indicates the assay's detection limit (reciprocal titer of 12.5). Values are geometric mean ± SD.
